# The Chemistry of Phenylimidotechnetium(V) Complexes with Isocyanides: Steric and Electronic Factors

**DOI:** 10.3390/molecules27238546

**Published:** 2022-12-04

**Authors:** Guilhem Claude, Laura Zeh, Maximilian Roca Jungfer, Adelheid Hagenbach, Joshua S. Figueroa, Ulrich Abram

**Affiliations:** 1 Institute of Chemistry and Biochemistry, Freie Universität Berlin, Fabeckstr. 34/36, 14195 Berlin, Germany; 2 Department of Chemistry and Biochemistry, University of California San Diego, La Jolla, CA 92093, USA

**Keywords:** technetium, phenylimides, isocyanides, ligand exchange, reactivity, DFT

## Abstract

Organometallic approaches are of ongoing interest for the development of novel functional ^99m^Tc radiopharmaceuticals, while the basic organotechnetium chemistry seems frequently to be little explored. Thus, structural and reactivity studies with the long-lived isotope ^99^Tc are of permanent interest as the foundation for further progress in the related radiopharmaceutical research with this artificial element. Particularly the knowledge about the organometallic chemistry of high-valent technetium compounds is scarcely developed. Here, phenylimido complexes of technetium(V) with different isocyanides are introduced. They have been synthesized by ligand-exchange procedures starting from [Tc(NPh)Cl_3_(PPh_3_)_2_]. Different reactivity patterns and products have been obtained depending on the steric and electronic properties of the individual ligands. This involves the formation of 1:1 and 1:2 exchange products of Tc(V) with the general formulae [Tc(NPh)Cl_3_(PPh_3_)(isocyanide)], *cis*- or *trans*-[Tc(NPh)Cl_3_(isocyanide)_2_], but also the reduction in the metal and the formation of cationic technetium(I) complex of the formula [Tc(isocyanide)_6_]^+^ when p-fluorophenyl isocyanide is used. The products have been studied by single-crystal X-ray diffraction and spectroscopic methods, including IR and multinuclear NMR spectroscopy. DFT calculations on the different isocyanides allow the prediction of their reactivity towards electron-rich and electron-deficient metal centers by means of the empirical SADAP parameter, which has been derived from the potential energy surface of the electron density on their potentially coordinating carbon atoms.

## 1. Introduction

**Figure 1:**
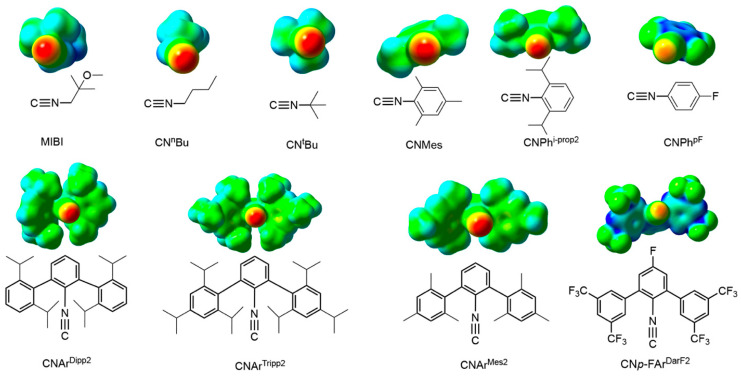
Isocyanides discussed in the present paper together with their electrostatic potential mapping (MO = 0.02; ρ_iso_ = 0.004) normalized to the potential boundaries of CNMe (7.478 × 10^−2^ [e/Å^3^]; blue = positive, red = negative) [11].

The impressive success story of the ^99m^Tc-Sestamibi (Cardiolite), a cationic hexakis(isocyanide) complex of technetium(I) with the ether-substituted MIBI ligand shown in Figure 1, dominates the chemistry of isocyanide complexes of technetium [1,2,3,4]. Thus, many such [Tc(CNR)_6_]^+^ complexes have been isolated and tested for their biological behavior [5,6,7]. ^99m^Tc is a metastable nuclear isomer, which practically emits the exclusively γ radiation of E_γ_ = 141 keV. Its short half-life of 6 h and the ready availability via a ^99^Mo/^99m^Tc isotope generator make this nuclide the workhorse in diagnostic nuclear medicine, with some 40 million procedures per year. This accounts for approximately 80% of all nuclear medical procedures and 85% of diagnostic scans in nuclear medicine worldwide [8].

The concentration of the ^99m^TcO_4_^−^ solutions, which are eluted from commercial ^99^Mo/^99m^Tc generators, is approximately on a nanomolar level. This is a clear advantage for the medicinal use of the prepared drugs since classical toxicity problems normally play no role in such dilute solutions. On the other hand, structural and spectroscopic investigations, which are important for the development and improvement of drugs, are largely prohibited by dilution. They are commonly performed using a second isotope of technetium: the long-lived ^99^Tc. ^99^Tc is a weak β^−^ emitter with a low β energy (E_βmax_ = 0.292 MeV) and a half-life of 2.1 × 10^5^ years. It is available in macroscopic amounts as one of the major products of nuclear fission and is isolated from spent nuclear fuel solutions. Its weak β radiation allows the handling of ^99^Tc compounds in milligram amounts in normal glassware, provided that general radiation protection protocols are applied.

**Scheme 1:**
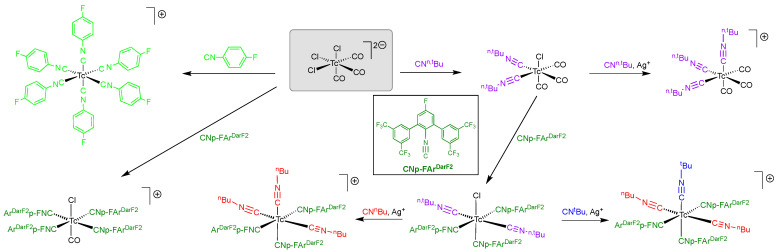
Reactions of carbonyltechnetium(I) complexes with differently substituted isocyanides [11].

Alkyl and aryl isocyanides are frequently regarded as structural and electronic surrogates for carbonyl ligands, even when a tendency is observed to act as stronger σ-donors and as weaker π-acceptors. Such a general description, however, is merely a rough approximation, and particularly the π-acceptor properties may be strongly influenced by the organic substituents of the ligands. Steric effects and the formal oxidation state of the metal ion will also influence the strength of the resulting metal–carbon bonds. Systematic studies about this point are rare, which is related to the fact that in many papers about the coordination chemistry of isocyanides, commercially available and stable ligands such as *tert-*butyl or cyclohexyl isocyanides are favorably used [9]. A more detailed assessment of the influence of electronic factors on the coordination behavior of highly substituted aryl isocyanides is available for a number of chromium(0) compounds, where a special role for aryl isocyanides with electron-withdrawing substituents was found [10]. Similar results were derived very recently for carbonyltechnetium(I) compounds, which react with differently substituted isocyanides in very different manners (Scheme 1) [11]. It became evident that simple alkyl isocyanides were not able to replace carbonyl ligands, while this was readily possible with aryl isocyanides which have a fluorine substituent in 4-position. Steric factors give control over the degree of the achieved ligand exchange. The influence of the substituents at the isocyanide ligands in such reactions has been reasoned with the DFT-derived electrostatic potential at the accessible surface of the corresponding isocyanide carbon atoms. The corresponding **S**urface-**A**veraged **D**onor **A**tom **P**otential (**SADAP**) parameter allowed predictions concerning the reactivity of the individual isocyanides with the regarded d^6^ systems in a synthetic and operationally convenient way [11]. It would now be interesting to see if the derived SADAP parameter is also suitable for electron-poor metal centers such as technetium(V) complexes, where no or almost no back-bonding to ligand orbitals should be expected.

There are only a very few examples of Tc(V) isocyanide complexes. Unlike the related rhenium compounds [12,13,14], oxotechnetium(V) complexes are readily reduced by isocyanides, and corresponding complexes could not (yet) be isolated. Thus, there exist only two nitridotechnetium(V) and phenylimidotechnetium(V) complexes with sterically encumbered terphenyl isocyanides [15]. Particularly the phenylimido compounds are interesting for a comparative study since the “NPh^2−^” ligand is isoelectronic to “O^2−^” but not prone to reduction. This may allow a comparison of the reaction behavior of different isocyanides with the {Tc(NPh)}^2−^ core, but also between technetium and rhenium, given that comparable rhenium complexes can also be synthesized. For both metals, there exist corresponding [M(NPh)Cl_3_(PPh_3_)_2_] complexes as potential starting materials [16,17], and ligand-exchange procedures starting from [Tc(NPh)Cl_3_(PPh_3_)_2_] have been used to prepare novel technetium(V) complexes with phosphines, dithiolenes, acetylacetones, or other ligands [18,19,20,21,22].

## 2. Results and Discussion

### 2.1. The Ligands

The results obtained during reactions of tricarbonyltechnetium(I) complexes and other metal centers with isocyanides strongly indicate that this class of ligands should not be regarded in the same undifferentiated way as carbonyl surrogates, although a few correlations between the properties of some isocyanides and their highest occupied molecular orbitals (HOMO) and lowest unoccupied molecular orbitals (LUMO) have been discussed in the past [23,24,25,26]. It has been found that electronic as well as steric effects strongly influence their coordination capabilities [10,11,12,13,14,15,27,28,29,30,31,32,33,34,35]. The electronic effects can best be described by a consideration of the electrostatic potentials located at the donor carbon atoms. Such parameters are generally accepted as tools for an evaluation of the nucleophilicity (electron-richness) or electrophilicity (electron-deficiency) of atoms or fragments of molecules [36,37,38]. With the intention to apply such measures also for the estimation of σ-donor/π-acceptor properties of isocyanides, we modeled several isocyanides by DFT calculations at the B3LYP/6-311++G* level. Details of the calculations are outlined in Ref. [11]. The final self-consistent field densities were used for the construction of mappings of the electrostatic potentials onto the density isosurfaces (MO = 0.02; *ρ* = 0.004). They revealed stunning substitution-dependent differences when normalized to the potential boundaries [e/Å³] of the intermediate donor CNMe. The corresponding electrostatic potential maps for the isocyanides discussed in this paper are shown in Figure 1, while corresponding maps for a large number of other isocyanides are published elsewhere [11].

Electron-deficient regions on the surface of the C ≡ N carbon atom could enable improved π-back donation (at least when bonded to electron-rich metal ions), while electron-rich regions on the surface of the same carbon atom would be responsible for a better σ-donation. Steric restraints on the donor carbon atom can be partially included in such an approach by averaging the obtained potential energies over the *accessible* surface of the potential donor atoms, which means the surface on the VdW boundary of a specific atom but not in the VdW boundary of another atom. Thus, we calculated the electrostatic and steric surface properties of the carbon atoms potentially involved in isocyanide-metal binding [38]. The sterically demanding isocyanides expectedly showed a less overall accessible surface area, while the less encumbered isocyanides had a larger overall accessible carbon surface. In a similar way, the rather electron-accepting isocyanides (partial π-acceptors) showed an increased positive surface area at the potential donor carbon atom compared to the rather σ-donor ligands, which had no positive surface exposure on their isocyanide carbon atom. 

The charge distribution on the surface also plays a crucial role in the interplay between π-acceptance and σ-donation. We introduced a simple descriptor for the overall donor/acceptor properties of isocyanides using the calculated potential energies (extrema and averaged values) on the vdW surface of the C ≡ N carbon atom and the exposed VdW surface area [11]. The resulting **S**urface-**A**veraged **D**onor **A**tom **P**otential (**SADAP**) sum parameter ($SADAP=\frac{EP_{min}+EP_{max}+AP}{ES_{pos}+ES_{neg}}$) corresponds to the averaged interaction energy of the C ≡ N carbon atom with positively and negatively charged moieties over the entire accessible surface. Table 1 contains the SADAP measures for the isocyanides discussed in the present paper, together with their components. The corresponding values of many more isocyanides are given as Supplementary Materials.

For the electron-rich (d^6^) carbonyltechnetium(I) complexes shown in Scheme 1, the calculated SADAP parameters nicely correlate with the observed reactivity of the compounds (e.g., the ability of certain isocyanides to replace carbonyl ligands) [11]. Isocyanides with progressively positive overall sum parameters replace CO ligands on the low-valent metal complexes, while those with negative sum parameters are σ-donors with predominantly negligible back-bonding properties. Consequently, no, or only a slow exchange of carbonyl ligands is observed with the latter group of isocyanides. It should, however, be noted that particularly the π-acceptor behavior of the isocyanides in such complexes (expressed by the ν_C_ ≡ _N_ IR frequencies in the different complexes) is also significantly influenced by the nature and number of co-ligands [11,39].

In comparison, we found that the HOMO and LUMO energies (or those of the respective lone-pair at carbon and the π*_CN_ orbitals), as well as the magnitude of the LP_C_-π*_CN_ gaps in the free ligands, do not reflect the reactivity well and, therefore, suggest the molecular-orbital-derived electrostatic surface potential parameter SADAP as a better descriptor for the reactivity compared to direct molecular orbital approaches. Since the above-mentioned considerations are exclusively ‘ligand-based’, we also calculated the electronic properties of some model complexes of the theoretical high-valent composition [Tc^VII^O_3_(CO/CNR)]^+^ and the low-valent composition [Tc^I^(CO)_5_(CO/CNR)]^+^ (R = Ph^F5^, Ph*^p-^*^F^, Ph, *^t^*Bu, Ar^DArF2^) with some representative isocyanides of considerably different SADAP parameters. Expectedly, for the fully oxidized technetium(VII) compounds, only σ–bonds were found; however, the number of electrons shared in the bond decreased in the non-intuitive order CO < CNPh^F5^ < CN Ph*^p-^*^F^ < CNPh < CN*^t^*Bu < CNAr^DArF2^. In contrast, the technetium atoms in the [Tc^I^(CO)_5_(CO/CNR)]^+^ cations show pronounced π-back donating behavior from two Tc lone-pairs to the two π*_CN_ orbitals with second-order perturbation energies between 8 and 14 kJ/mol in the order of CO > CNPh^F5^ > CNAr^DArF2^ > CN Ph^pF^ > CNPh > CN*^t^*Bu, while the ordering of the number of shared electrons in the σ–bonds is CO > CNPh^F5^ > CN Ph*^p-^*^F^ > CNPh > CNAr^DArF2^ > CN*^t^*Bu. It should be noted that only the second-order perturbation energies somewhat reflect the reactivity predictions made by SADAP, albeit at much higher computational costs, and common convergence problems arise—especially for large ligands. The corresponding parameters for the model compounds and the HOMO/LUMO information of some representative complexes are given in the Supplementary Materials.

Having in mind the nice correlation between the SADAP measures of Table 1 and the reactivity of the corresponding isocyanides with carbonyltechnetium(I) compounds [11], it should be interesting to compare the influence of steric and electronic parameters of the isocyanide ligands on the coordination to electron-poor metal ions such as the phenylimidotechnetium(V) core of the present study.

### 2.2. Reactions of [Tc(NPh)Cl_3_(PPh_3_)_2_] with Alkyl and (Alkyl-Substituted) Aryl Isocyanides

[Tc(NPh)Cl_3_(PPh_3_)_2_] (**1**) has been proven to be a suitable precursor material for the synthesis of other phenylimido complexes of technetium [18,19,20,21,22]. The compound is sufficiently soluble in solvents such as CHCl_3_ or CH_2_Cl_2_ and is stable enough to resist prolonged heating in organic solvents. This is unlike the behavior of the rhenium analog [Re(NPh)Cl_3_(PPh_3_)_2_], which is practically insoluble in all common organic solvents and is, thus, only partially suitable as a starting material for ligand exchange reactions [40]. The solubility of such compounds can be increased by the introduction of a fluorine atom at the imido ligand, as has been demonstrated by the synthesis of [Re{NC_6_H_4_(*p*-F)}Cl_3_(PPh_3_)_2_], and its use as a precursor for the synthesis of corresponding complexes with β-diketonates [40,41]. Such an approach is not suitable for related technetium compounds since the corresponding complexes with the *p*-substituted {=NC_6_H_4_(*p*-F)}^2−^ or {=NC_6_H_4_(*p*-CF_3_)}^2−^ ligands undergo rapid hydrolysis, which is followed by a reduction in the metal and the formation of the technetium(IV) complex [TcCl_4_(PPh_3_)_2_] [41].

**Scheme 2:**
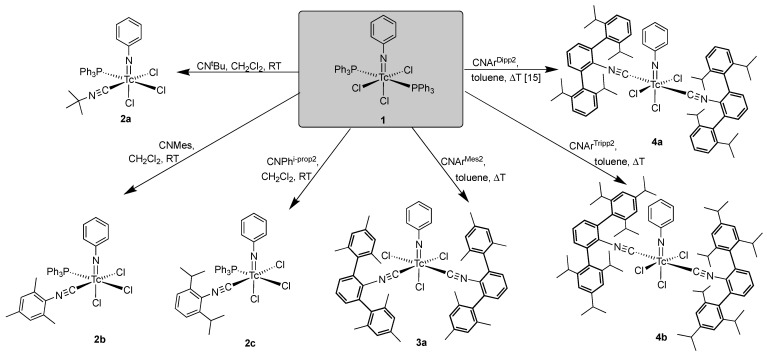
Reactions of [Tc(NPh)Cl_3_(PPh_3_)_2_] with alkyl and (alkyl-substituted) aryl isocyanides.

Fortunately, such decompositions are not common for the unsubstituted phenylimido complex, and [Tc(NPh)Cl_3_(PPh_3_)_2_] (**1**) reacts readily with alkyl isocyanides (e.g., CN^*t*^Bu) and small alkyl-substituted phenyl isocyanides such as CNMes or CNPh^i-prop2^ under replacement of one triphenylphosphine ligand and the formation of complexes of type **2**. The reactions proceed already at room temperature in solvents such as CH_2_Cl_2_ or CHCl_3_ (Scheme 2). The use of an excess of the isocyanides or heating does not result in the formation of products with two or more of such CNR ligands but causes problems during the isolation of the products in crystalline form by the presence of excess ligand and/or its decomposition products.

Interestingly, another course of the reaction is observed when [Tc(NPh)Cl_3_(PPh_3_)_2_] is treated with an excess of the sterically encumbered m-terphenylisocyanides CNAr^Mes2^, CNAr^Dipp2^, or CNAr^Tripp2^. In such cases, bis–complexes are formed under the replacement of both PPh_3_ ligands. These reactions require elevated temperatures, but we found no evidence for the formation of 1:1 ligand-exchange products under milder conditions or with the addition of only one equivalent of the terphenylisocyanides.

The observed difference in the reactivity goes along with the electrostatic potentials located at the carbon donor atoms of the isocyanides (Figure 1) and the clear differences found for the derived SADAP values calculated for the individual ligands (−3.38 and −3.28 for CNAr^Tripp2^ and CNAr^Dipp2^ and values between −2.28 and −2.15 for CN^t^Bu, CNMes, and CNPh^i-prop2^, Table 1). This means that the electron-rich isocyanides with a pronounced σ-donor and diminished π-acceptor behavior (such as CNAr^Tripp2^ and CNAr^Dipp2^) show a higher tendency for ligand exchange reactions at the electron-poor d^2^ metal centers of the technetium(V) complexes of the present study. The domination of the ‘σ-donation’ comes not completely unexpected and reflects a reversed behavior compared to the carbonyltechnetium(I) complexes of Scheme 1, where the π-back donation from the electron-rich d^6^ system and the competition with the good π-acceptor CO plays a major role [11]. Thus, ligands with a progressively positive overall sum parameter easily and rapidly replace CO ligands, while those with negative sum parameters have predominantly σ-donating properties, which rule the ligand-exchange behavior with electron-deficient metal centers as in phenylimidotechnetium(V) complexes. The absence of π-back donation is also reflected by the ν_CN_ IR frequencies. They appear in the Tc(V) complexes generally at higher wavenumbers than in the spectra of the uncoordinated ligands.

The [Tc(NPh)Cl_3_(PPh_3_)(isocyanide)] complexes **2** are green crystalline solids, while the bis–complexes **3a** and **4** are yellow-green. Both types of complexes are fairly soluble in CH_2_Cl_2_ or CHCl_3_ and insoluble in hydrocarbons. As solids, they are indefinitely stable at ambient temperatures in the air. As d^2^ systems with a multiply bonded phenylimido ligand, the novel compounds are diamagnetic and give well-resolved ^1^H spectra (see Experimental and Supplementary Materials). Their limited solubility, however, prevents the measurement of the ^13^C NMR spectra with sufficient quality.

**Figure 2:**
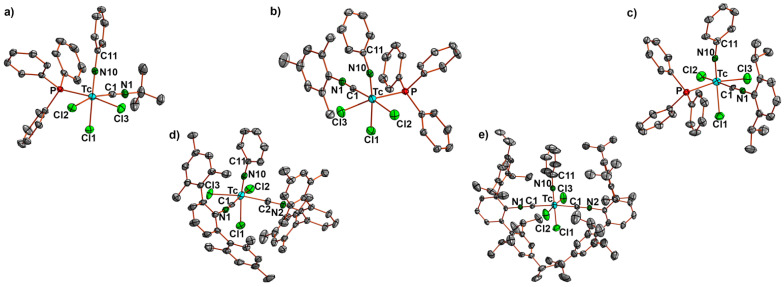
Ellipsoid representations of the molecular structures of (**a**) [Tc(NPh)Cl_3_(PPh_3_)(CN^t^Bu)] (**2a**), (**b**) [Tc(NPh)Cl_3_(PPh_3_)(CNMes)] (**2b**), (**c**) [Tc(NPh)Cl_3_(PPh_3_)(CNPh^i-prop2^)] (**2c**), (**d**) *cis*-[Tc(NPh)Cl_3_(CNAr^Mes2^)_2_] (**3a**) and (**e**) *trans*-[Tc(NPh)Cl_3_(CNAr^Tripp2^)_2_] (**4b**).

Single crystals of the complexes suitable for X-ray diffraction were obtained from CH_2_Cl_2_/hydrocarbon mixtures. Ellipsoid plots of the molecular structures are shown in Figure 2. Table 2 contains some selected bond lengths and angles. The Tc-N10 bond lengths are between 1.692(5) and 1.725(6) Å, which is clearly in the range of technetium–nitrogen double bonds and agreement with the values of other phenylimido complexes of technetium [15,16,17,18,19,20,21,22,41]. The Tc-N10-C11 bonds of the mono-substituted complexes **2** are slightly bent away from the bulky PPh_3_ ligand. A similar effect is observed for the bis-substituted *cis*-[Tc(NPh)Cl_3_(CNAr^Mes2^)_2_] (**3a**), in which the two bulky isocyanide ligands are coordinated in *cis* positions to each other, and a slightly bent arrangement of the {NPh}^2−^ ligand lowers the steric stress at the central part of the molecule.

It is interesting to note that the lower steric bulk of the CNAr^Mes2^ ligand allows the electronically favored *cis* coordination, while the CNAr^Dipp2^ and CNAr^Tripp2^ ligands are directed into the *trans*-conformation in compounds **4a** and **4b**. Similar findings are reported for nitridotechnetium(V) and oxidorhenium(V) complexes [14,15]. Notably, for the latter group of compounds, even the mixed–isocyanide complex [ReOCl_3_(CNAr^Mes2^)(CNAr^Dipp2^)]^−^ allows *cis*-coordination for the two isocyanides. The lower steric bulk of CNAr^Mes2^ might also allow for the more dynamic behavior of this ligand. Although we only isolated compound **3a** in its *cis* conformation, the NMR spectra of the corresponding reaction mixtures and solutions of **3** indicate a kind of fluxional behavior of this complex in solution with the formation of additional species. This can be regarded as a hint for a potential isomerization but also for the formation of mono- or tris-isocyanide complexes that cannot completely be excluded. Sterically, the latter option might also be possible since a corresponding dicarbonylmanganese(I) complex, [Mn(CO)_2_Br(CNAr^Mes2^)_3_] hosts three CNAr^Mes2^ ligands in meridional positions [32].

### 2.3. Reactions of [Tc(NPh)Cl_3_(PPh_3_)_2_] with Fluorine-Substituted Aryl Isocyanides

**Scheme 3:**
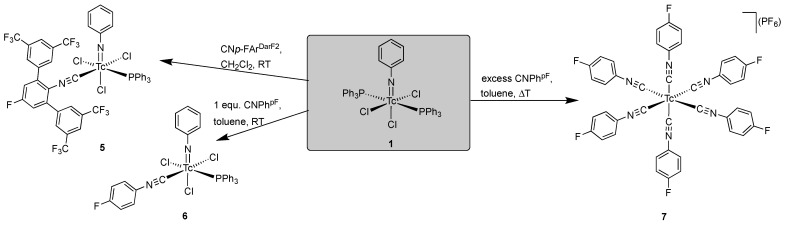
Reactions of [Tc(NPh)Cl_3_(PPh_3_)_2_] with CN*p*-FAr^DarF2^ and CNPh^pF^.

A ligand with particularly interesting properties is CN*p*-FAr^DarF2^. It combines a fluorine-substituted central phenyl ring with two bulky bis(trifluoromethyl)phenyl substituents. It is extremely electron-deficient at the isocyanide carbon atom, which makes it a good π-acceptor, and even allows for the exchange of carbonyl ligands [11,35]. Unlike the reactions with the d^6^ systems in Scheme 1, these properties should be without relevance for reactions with the electron-deficient technetium(V) complexes. Additionally, during a ligand exchange reaction with [Tc(NPh)Cl_3_(PPh_3_)_2_], only one CN*p*-FAr^DarF2^ ligand enters the coordination sphere of the metal, and pale green crystals of [Tc(NPh)Cl_3_(PPh_3_)(CN*p*-FAr^DarF2^)] (**5**) are formed (Scheme 3).

Attempted reactions with an excess of the ligand did not result in the formation of technetium complexes with more CN*p*-FAr^DarF2^ ligands. Since steric reasons seem to be irrelevant for this result and technetium complexes with up to four CN*p*-FAr^DarF2^ ligands in their coordination sphere are known [11,35], we attribute the observed reaction behavior to electronic reasons. Such an assumption is supported by the SADAP parameter of CN*p*-FAr^DarF2^, which is the most positive in Table 1 and suggests a predominantly poor σ-donor behavior. Nevertheless, the ν_CN_ frequency in complex **5** also appears at a higher wavenumber (2176 cm^−1^) than in the uncoordinated isocyanide. 

**Figure 3:**
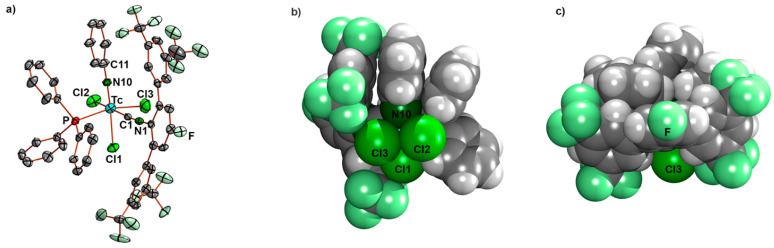
(**a**) Ellipsoid representation of the molecular structure of [Tc(NPh)Cl_3_(PPh_3_)(CN*p*-FAr^DarF2^)] (**5**) and illustration of the steric bulk caused by the isocyanide and PPh_3_ ligands by (**b**) A view along the phenylimido plane and (**c**) A view along the isocyanide bond. Selected bond lengths and angles: Tc-N10 1.710(4) Å, Tc-Cl1 2.434(2) Å, Tc-Cl2 2.398(2) Å, Tc-Cl3 2.389(2) Å, Tc-C1 2.009(5) Å, C1-N1 1.176(6) Å, N10-Tc-Cl1 165.5(2)°.

Single crystals of [Tc(NPh)Cl_3_(PPh_3_)(CN*p*-FAr^DarF2^)] (**5**) suitable for X-ray diffraction were obtained by slow diffusion of *n*-hexane into a solution of the complex in CH_2_Cl_2_. An ellipsoid representation of the molecular structure of **5** is depicted in Figure 3a, while Figure 3b,c illustrates the steric bulk caused by the *cis*-coordinated isocyanide and PPh_3_ ligands. The bonding situation around the technetium atom of complex **5** is expectedly very similar to those found for complexes **2a**, **2b,** and **2c** with a Tc-N10 double bond of 1.710(4) Å and a slightly bent N10-Tc-Cl1 axis.

In contrast to the compounds of type **2**, solutions of [Tc(NPh)Cl_3_(PPh_3_)(CN*p*-FAr^DarF2^)] (**5**) are not infinitely stable. A gradual decomposition of **5** becomes evident when solutions of the compound are heated. This can be concluded from the appearance of novel ^19^F NMR signals in such solutions (see Supplementary Materials).

**Figure 4:**
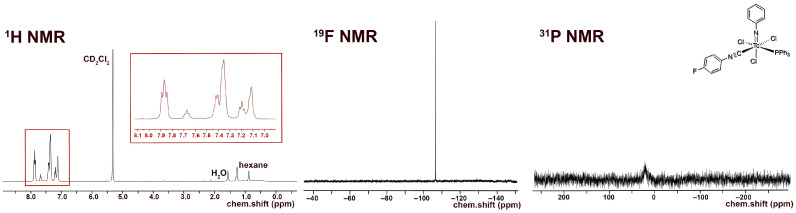
^1^H, ^19^F and ^31^P NMR spectra of [Tc(NPh)Cl_3_(PPh_3_)(CNPh^pF^)] (**6**) in CD_2_Cl_2_.

Even more unstable is the ligand exchange product of [Tc(NPh)Cl_3_(PPh_3_)_2_] with CNPh^pF^: [Tc(NPh)Cl_3_(PPh_3_)(CNPh^pF^)] (**6**). This ligand has been chosen as a sterically unencumbered mimic for CN*p*-FAr^DarF2^. The isocyanide carbon atom of CNPh^pF^ is also extremely electron deficient with only a small negative SADAP parameter of −1.25 (Table 1), for which a reactivity similar to CN*p*-Far^DarF2^ or alkyl or simple aryl isocyanides should be expected. Indeed, the 1:1 ligand exchange product **6** could be isolated, but the synthesis had to be performed under mild conditions, and quick precipitation of the product was required to obtain a pure compound. [Tc(NPh)Cl_3_(PPh_3_)(CNPh^pF^)] is a pale green, microcrystalline complex that is readily soluble in CH_2_Cl_2_. The ^1^H and ^19^F NMR spectra of reasonable quality can be recorded for the pure compound from fresh solutions of the complex in this solvent (Figure 4). A well-resolved ^31^P NMR spectrum could not be obtained. Such a behavior is not unusual for (low-symmetric) phosphine complexes of technetium and is commonly explained by scalar couplings with the large quadrupole moment of ^99^Tc (Q = −0.19 Å × 10^−28^ m^2^) [42,43]. Such couplings can cause extreme line-broadenings, which make the resolution of ^31^P signals frequently impossible [44,45,46,47], which is also observed for the complexes of type **2**. An ongoing decomposition of the compound in the solution becomes evident by the detection of an increasing number of ^19^F NMR signals within one day in the solution. The infrared spectrum of complex **6** shows the ν_CN_ band at 2186 cm^−1^, which is at a higher wavenumber than that of the uncoordinated ligand (2129 cm^−1^) and confirms the assumption that a potential π-acceptor behavior of CNPh^pF^ is not significant for the phenylimdo complexes of technetium(V).

A completely unexpected product was found for the reaction of [Tc(NPh)Cl_3_(PPh_3_)_2_] with an excess of CNPh^pF^ in boiling toluene. The reaction of compound **1** with CNPh^pF^ under such conditions gives the Tc(I) cation [Tc(CNPh^pF^)_6_]^+^. Such behavior of CNPh^pF^ has been observed before during reactions with (NBu_4_)[Tc^I^_2_(CO)_6_Cl_3_] or [Tc^I^(CO)_3_(CN^t^Bu)_2_Cl] [11]. However, in the latter cases, it could readily be explained by the activity of the fluorine-substituted ligand as a strong π-acceptor. In the case of the technetium(V) complexes of the present study, this does not apply. However, having in mind the inherent instability of the 1:1 exchange product [Tc(NPh)Cl_3_(PPh_3_)(CNPh^pF^)], we regard it as highly probable that in the course of the reaction, the technetium–nitrogen bond is cleaved, which should result in a rapid reduction in the metal to low oxidation states. Consequently, the strong π-acceptor CNPh^pF^ will gradually dominate the chemistry in the system, and the formation of the stable [Tc(CNPh^pF^)_6_]^+^ cation becomes unavoidable.

The hexakis complex can be isolated as PF_6_^−^ salt in the form of colorless microcrystals. [Tc(CNPh^pF^)_6_](PF_6_) (**7**) is infinitely stable in the air. It shows a narrow (ν_1/2_ = 42 Hz) ^99^Tc NMR signal at −1886 ppm, which comes close to the value of [Tc(CNPh)_6_]^+^ in CDCl_3_ (−1889 ppm), but is appreciably de-shielded relative to the values found for hexakistechnetium(I) cations with alkyl isocyanides (−1914 to −1964 ppm) [45]. The infrared spectrum of **7** displays the ν_CN_ band at 2087 cm^−1^, which corresponds to a bathochromic shift of ca. 30 cm^−1^ relative to the value in uncoordinated CNPh^pF^ and indicates a marked π-back-donation to the isocyanide ligand in this technetium(I) complex.

## 3. Materials and Methods

Unless otherwise stated, reagent-grade starting materials were purchased from commercial sources and either used as received or purified by standard procedures. The solvents were dried and deoxygenated according to standard procedures. [Tc(NPh)Cl_3_(PPh_3_)_2_] (1), CNAr^Tripp2^ and [Tc(NPh)Cl_3_(CNAr^Dipp2^)_2_] (**4a**) were prepared by procedures in the literature [10,15,16]. The syntheses of CN*p*-FAr^DarF2^, CNPh^pF^, CNMes, and CNPh^i-prop2^ were performed by modified procedures from the literature [9,11]. The NMR spectra were recorded with JEOL 400 MHz ECS or ECZ multinuclear spectrometers. The values given for the ^99^Tc chemical shifts are referenced to potassium pertechnetate in water. IR spectra were recorded with a Shimadzu FTIR 8300 spectrometer as KBr pellets. Intensities are classified as vs. = very strong, s = strong, m = medium, w = weak, vw = very weak, and sh = shoulder.

### 3.1. Radiation Precautions

^99^Tc is a long-lived, weak β^−^ emitter (E_max_ = 0.292 MeV). Normal glassware provides adequate protection against weak beta radiation when milligram amounts are used. Secondary X-rays (bremsstrahlung) play a significant role only when larger amounts of ^99^Tc are handled. All manipulations were performed in a laboratory approved for the handling of radioactive materials.

### 3.2. Syntheses

The general procedure for the [Tc(NPh)Cl_3_(PPh_3_)(CNR)] Complexes **2**: [Tc(NPh)Cl_3_(PPh_3_)_2_] (**1**) (41 mg, 0.05 mmol) was dissolved in CH_2_Cl_2_ (5 mL). The corresponding isocyanide (0.055 mmol) was added, and the solution was stirred for 10 min at room temperature. Volatiles were removed under reduced pressure, and the residue was resuspended in Et_2_O (5 mL) and filtered. The obtained solid was washed with Et_2_O (3 × 5 mL) and then dried under reduced pressure. Single crystals suitable for X-ray diffraction were obtained by the slow diffusion of n-hexane into solutions of the complexes in CH_2_Cl_2_. The obtained crystals were filtered, washed with a small amount of Et_2_O, and dried under reduced pressure.

*[Tc(NPh)Cl_3_(PPh_3_)(CN^t^Bu)]* (**2a**): Green needles. Yield: 15 mg, 47%. IR (cm^−1^): 3057 (w), 2984 (w), 2918 (w), 2207 (s, ν_C≡N_), 1437 (s), 1191 (m), 1092 (m), 749 (m), 695 (s), 525 (s). ^1^H NMR (CD_2_Cl_2_, ppm): δ = 7.82 (m_c_, 6H, *o*-H (PPh_3_)), 7.65 (t, *J* = 7.46 Hz, 1H, *p*-H (arom. NPh)), 7.46 (m_c_, 3H, *p*-H (PPh_3_)), 7.39 (m_c_, 6H, *m*-H (PPh_3_)), 7.30 (d, *J* = 7.84 Hz, 2H, *o*-H (arom. NPh)), 7.17 (t, *J* = 7.75 Hz, 2H, *m*-H (arom. NPh)), 1.38 (s, 9H, (CH_3_)_3_).

*[Tc(NPh)Cl_3_(PPh_3_)(CNMes)]* (**2b**): Green needles. Yield: 10 mg, 28%. IR (cm^−1^): 3057 (w), 2920 (w), 2187 (s, ν_C≡N_), 1480 (w), 1435 (m), 1310 (w), 1092(m), 747 (m), 693 (s), 523 (s). ^1^H NMR (CD_2_Cl_2_, ppm): 7.87 (m_c_, 6H, *o*-H(PPh_3_)), 7.66 (t, *J* = 7.47 Hz, 1H, *p*-H (arom. NPh)), 7.41–7.31 (m, 11H, *m*-/*p*-H(PPh_3_), *o*-H (arom. NPh)), 7.18 (t, *J* = 7.77 Hz, 2H, *m*-H (arom. NPh)), 6.87 (s, 2H, *m*-H (arom. CNMes)), 2.32 (s, 3H, *p*-CH_3_ (CNMes)), 1.94 (s, 6H, *o*-CH_3_ (CNMes)).

*[Tc(NPh)Cl_3_(PPh_3_)(CNPh^i-prop2^)]* (**2c**): Green-yellow, dichroic needles. Yield: 26 mg, 70%. IR (cm^−1^): 3055 (w), 2695 (m), 2922 (w), 2183 (s, ν_C≡N_), 1572 (m), 1477 (m), 1433 (s), 980 (w), 804 (w), 746 (m), 692 (m), 525 (m). ^1^H NMR (CD_2_Cl_2_, ppm): 7.89 (m_c_, 6H, o-H(PPh_3_)), 7.69 (t, *J* = 8.0 Hz, 1H, *p*-H (arom. NPh)), 7.43–7.29 (m, 12H, *m*-/*p*-H (PPh_3_), H(CNPh*^i-^*^prop2^)), 7.20 (t, *J* = 8.0 Hz, 2H, *m*-H (arom. NPh)), 7.14 (d, *J* = 7.7 Hz, 2H, *o*-H (arom. NPh)), 2.68 (h, *J* = 6.8 Hz, i-prop CH), 0.97 (d, *J* = 7.0 Hz, i-prop CH_3_), 0.89 (d, *J* = 7.0 Hz, i-prop CH_3_).

Subsequently, *cis-[Tc(NPh)Cl_3_(CNAr^Mes2^)_2_]* (**3a**): [Tc(NPh)Cl_3_(PPh_3_)] (**1**) (82 mg, 0.1 mmol) was suspended in toluene (5 mL). CNAr^Mes2^ (68 mg, 0.2 mmol) was added, and the reaction mixture was heated under reflux for one hour. It became dark green and homogenous upon heating. The resultant solution was slowly evaporated at 5 °C. After one day, the first crop of a few yellow-green needles (compound **3a**) suitable for X-ray diffraction were obtained and analyzed by IR spectroscopy. Upon further evaporation of the solvent, more of the aforementioned needles was obtained along with other green crystals of different shapes, which were not suitable for X-ray diffraction. They were filtered off and washed with small amounts of n-pentane and studied by ^1^H NMR spectroscopy. Three sets of resonances were observed in the methyl region, suggesting the presence of at least three isomers which could be cis/trans-[Tc(NPh)Cl_3_(CNAr^Mes2^)_2_] or cis/trans-[Tc(NPh)Cl_3_(PPh_3_)(CNAr^Mes2^)]. **3a**: Yellow-green needles. IR (cm^−1^): 3058 (w), 2919 (m), 2851 (w), 2178 (s, ν_C≡N_), 1572 (m), 1433 (m), 1308 (w), 1094 (w), 845 (w), 749 (w), 695 (m), 521 (m). The isomeric mixture was of [Tc(NPh)Cl_3_(CNAr^Mes2^)_2_] and [Tc(NPh)Cl_3_(PPh_3_)(CNAr^Mes2^)]: IR (cm^−1^): 3058 (w), 2919 (m), 2851 (w), 2178 (s, ν_C≡N_), 1572 (m), 1433 (m), 1308 (w), 1094 (w), 845 (w), 749 (w), 695 (m), 521 (m). ^1^H NMR (CD_2_Cl_2_, ppm): 7.78–6.58 (m, aryl), 2.20 (s, CH_3_), 2.11 (s, CH_3_), 2.05 (s, CH_3_), 2.02 (s, CH_3_), 1.96 (s, CH_3_), 1.94 (s, CH_3_).

*trans-[Tc(NPh)Cl_3_(CNAr^Tripp2^)_2_]* (**4b**): [Tc(NPh)Cl_3_(PPh_3_)] (**1**) (41 mg, 0.05 mmol) was suspended in toluene (5 mL). CNAr^Tripp2^ (51 mg, 0.1 mmol) was added, and the reaction mixture was heated under reflux for one hour. It became slightly red and homogenous upon heating. The resultant solution was slowly evaporated at 5 °C. After five days, green-yellow crystals suitable for X-ray diffraction were obtained. They were filtered off and washed with small amounts of cold MeOH and n-pentane, and then dried under a reduced pressure. Yield: 39 mg, 72%. IR (cm^−1^): 3057 (w), 2957 (s), 2866 (s), 2184 (s, ν_C≡N_), 1607 (m), 1576 (m), 1570 (m), 1461 (s), 1439 (s), 1362 (s), 1316 (m), 1191 (m), 1121 (m), 1071 (w), 943 (w), 874 (s), 807 (m), 722 (m), 697 (s), 543 (s). ^1^H NMR (CD_2_Cl_2_, ppm): 7.73 (t, *J* = 7.33 Hz, 1H, p-H (arom. NPh)), 7.50 (t, *J* = 6.8 Hz, 2H, CNAr^Tripp2^), 7.41 (d, *J* = 7.46 Hz, 2H, o-H (arom. NPh)), 7.26 (d, *J* = 7.46 Hz, 4H, CNAr^Tripp2^), 7.14 (t, *J* = 6.68 Hz, 2H, m-H (arom. NPh)), 6.92 (s, 8H, CNAr^Tripp2^), 2.83 (h, *J* = 6.1 Hz, 4H, (i-prop CH)), 2.38 (h, *J* = 6.3 Hz, 8H, (i-prop CH)), 1.32 (d, *J* = 6.52 Hz, 24H, (i-prop CH_3_)), 1.01 (d, *J* = 6.0 Hz, 24H, (i-prop CH_3_)), 0.96 (d, *J* = 6.0 Hz, 24H, (i-prop CH_3_)).

*[Tc(NPh)Cl_3_(PPh_3_)(CNp-FAr^DarF2^)]* (**5**): [Tc(NPh)Cl_3_(PPh_3_)_2_] (**1**) (41 mg, 0.05 mmol) was dissolved in CH_2_Cl_2_ (5 mL). CNp-FAr^DarF2^ (30 mg, 0.055 mmol) was added, and the dark green solution was stirred for 20 min at room temperature. A pale green solid was precipitated by the addition of an excess of n-hexane (approximately 30 mL). The immediately formed precipitate was washed with pentane and a small amount of diethyl ether, redissolved in CH_2_Cl_2_ (1 mL), and overlayered with n-hexane. Pale green columns were formed together with brown oil. The crystals were separated and washed with pentane alongside a small amount of diethyl ether, and the crystallization procedure was repeated in the described way. The resultant single crystals were suitable for X-ray diffraction. Pale green needles. Yield: 22 mg, 40%. IR (cm^−1^): 3057 (w), 2176 (vs, ν_C≡N_), 1482 (w), 1435 (m), 1364 (m), 1279 (s), 1179 (s), 1135 (s), 1092 (m), 905 (w), 743 (m), 701 (m), 693 (m) 520 (m). ^1^H NMR (CD_2_Cl_2_, ppm): 7.90 (s, 4H, CNp-FAr^DarF2^), 7.61 (s, 2H, CNp-FAr^DarF2^), 7.55 (t, *J* = 7.1 Hz, 1H, p-H (arom. NPh)), 7.46 (t, *J* = 8.4 Hz, 6H, o-H (PPh_3_)), 7.31 (t, *J* = 6.9 Hz, 3H, p-H (PPh_3_)), 7.25 (d, J = 7.7 Hz, 2H, m-H (CNp-FAr^DarF2^)), 7.16 (t, *J* = 6.0 Hz, 6H, m-H (PPh_3_)), 6.89 (t, *J* = 7.2 Hz, 2H, m-H (arom. NPh)), 6.71 (d, *J* = 6.9 Hz, 2H, o-H (arom. NPh)). ^19^F NMR (CD_2_Cl_2_, ppm): −65.0 (s, 12F, m,m’-CF_3_ (CNp-FAr^DarF2)^), −107.9 (s, 1F, p-F (CNp-FAr^DarF2^)).

*[Tc(NPh)Cl_3_(PPh_3_)(CNPh^F^)]* (**6**): 100 µL of a solution prepared from CNPh^pF^ (160 µL) and toluene (940 µL was added to a suspension of [Tc(NPh)Cl_3_(PPh_3_)_2_] (**1**) (41 mg, 0.05 mmol) in CH_2_Cl_2_ (5 mL). The mixture was gently heated and held at a temperature of 30 °C until the reaction mixture became homogenous (approximately 2 min). Then, *n*-hexane (20 mL) was immediately added, which resulted in the formation of a pale green precipitate. The obtained solid (pure compound **6** with some incorporated *n*-hexane) was filtered off, washed with diethyl ether alongside *n*-hexane, and then dried under reduced pressure. Complex **6** is stable as a solid but decomposes at room temperature in solvents such as dichloromethane or acetone. Yield: 20 mg, 57%. IR (cm^−1^): 3422 (br), 3058 (w), 2923 (w), 2186 (vs, ν_C≡N_), 1570 (w), 1499 (s), 1435 (m), 1239 (w), 1092 (m), 990 (w), 841 (m), 749 (m), 697 (s), 521 (s). ^1^H NMR (CD_2_Cl_2_, ppm): 7.80 (m_c_, 6H, o-H(PPh_3_)), 7.68 (t, *J* = 8.0 Hz, 1H, *p*-H (arom. NPh)), 7.46–7.31 (m, 11H), 7.20 (t, *J* = 8.0 Hz, 2H, *m*-H (arom.)), 7.17–7.07 (m, 4H), 1.27 (CH_2_, 0.2 n-hexane), 0.88 (CH_3_, 0.2 n-hexane). ^19^F NMR (CD_2_Cl_2_, ppm): −106.5.

*[Tc(CNPh^pF^)_6_](PF_6_)* (**7**): [Tc(NPh)Cl_3_(PPh_3_)_2_] (**1**) (41 mg, 0.05 mmol) was suspended in toluene (5 mL). CNPh^pF^ (45.9 μL, 0.5 mmol) was added, and the solution was heated under reflux for one hour. The reaction mixture became homogeneous upon heating and changed its color to pale yellow within the first ten minutes. Then, a solid started to precipitate. The reaction mixture was cooled to room temperature and filtered. The obtained solid was washed with a small amount of toluene and redissolved in MeOH. NH_4_(PF_6_) (0.5 g) was dissolved in a water/MeOH mixture (5 mL, 1:1) which was added. A colorless solid precipitated, which was filtered off and washed sequentially with water, MeOH, and Et_2_O. Yield: 12 mg, 26%. IR (cm^−1^): 2918 (w), 2087 (s, ν_C≡N_), 1501 (m), 1235 (m), 1154 (m), 836 (m), 558 (w). ^1^H NMR (CD_2_Cl_2_, ppm): 7.46 (m_c_, 12H, o-H (CNPh^pF^)), 7.16 (t, *J* = 8.5 Hz, 12H, m-H (CNPh^pF^)). ^19^F NMR (CD_2_Cl_2_, ppm): −73.4 (d, ^1^*J* (^19^F-^31^P) = 670 Hz, 6F, PF_6_), −109.2 (s, 6F, (CNPh^pF^)). ^99^Tc NMR (CD_2_Cl_2_, ppm): −1886 (s, ν_1/2_ = 42 Hz).

### 3.3. X-ray Crystallography

The intensities for the X–ray determinations were collected on the STOE IPDS II instrument with Mo Kα radiation. The space groups were determined by the detection of systematic absences. Absorption corrections were carried out by multi-scan or integration methods [48]. The structure solution and refinement were performed with the SHELX program package [49,50]. Hydrogen atoms were derived from the final Fourier maps and were refined or placed at calculated positions and treated with the ‘riding model’ option of SHELXL.

Compound **2c** crystallizes in the non-centrosymmetric space group Pca2_1_. The absolute structure has been determined with a Flack parameter of 0.06(6). Compound **3** co-crystallizes with CH_2_Cl_2_ molecules in the large voids between the complex molecules. One molecule of dichloromethane could be located and refined. A sum of 102 e, which corresponds to another 2.5 molecules of CH_2_Cl_2_, has been ‘removed’ from the final Fourier map by means of a solvent mask of OLEX2 [51]. The positional disorder of isopropyl or CF_3_ groups has been addressed during the refinement of the crystal structures of compounds **4b** and **5**. Details can be inspected in the Supplementary Materials and/or the corresponding cif files.

The representation of molecular structures was conducted using the program DIAMOND 4.2.2 [52].

### 3.4. Computational Details

DFT calculations were performed on the high-performance computing systems of the Freie Universität Berlin ZEDAT (Curta) using the program package GAUSSIAN 16 [53,54]. The gas phase geometry optimizations were performed using initial coordinates generated using GAUSSVIEW and Avogadro [55,56]. The calculations were performed with the hybrid density functional B3LYP [57,58,59]. The Stuttgart relativistic small core basis set with its associated pseudo potential has been used for Tc [60,61]. The 6-311++G** basis set was used to model all other elements [62,63,64,65,66]. All basis functions, as well as the ECPs, were obtained from the EMSL database [67]. The surface properties module of the multifunctional wave-function analyzer MultiWFN was used for the calculation of the surface properties [38,68].

## 4. Conclusions

Using the Tc-phenylimido core, high-oxidation state isocyanide complexes of technetium(V) can be prepared and isolated as stable molecular species. [Tc(NPh)Cl_3_(PPh_3_)_2_] has been proven to be a suitable starting material for such reactions. One or both of the PPh_3_ ligands can be replaced by isocyanides depending on the electronic properties and the steric bulk of the incoming ligands. A DFT-based sum parameter (SADAP) describing the electrostatic potential at the isocyanide carbon atom is a good measure to predict the reactivity of isocyanides with metal ions. In contrast to low-valent technetium complexes, where electron-deficient isocyanides (less negative or positive SADAP parameters) show the highest exchange rates, reactions with electron-rich isocyanides such as CNAr^Mes2^ or CNAr^Tripp2^ (ligands with very negative SADAP parameters) form bis–complexes with the d^2^ systems under study. On the contrary, ligands with a lower electron density located at the isocyanide carbon atoms prefer the formation of 1:1 complexes. Such a behavior correlates with the σ-donor and π-acceptor properties of the ligands in the formed complexes in a way that for the complex formation with the electron-poor {Tc^V^(NPh)}^3−^ core, the σ-donor abilities of the ligands clearly dominate. This is also reflected by the positions of the ν_CN_ bands in the IR spectra of the complexes (Table 3). They are all found at higher wavenumbers relative to the spectra of the uncoordinated ligands. 

A considerable π-acceptor behavior (also reflected by a bathochromic shift of the corresponding ν_CN_ band) is only observed when the metal ion is reduced as a consequence of the cleavage of the technetium–nitrogen double bond. Such a reaction pathway has been observed for CNPh^pF^.

## Figures and Tables

**Table 1:** Calculated electrostatic potential surface properties of the isocyanide carbon atom at the VdW boundary for structures optimized at the B3LYP/6-311++G** level. Surface properties were evaluated at ρ = 0.001 level using an electrostatic potential map basis with a grid-point spacing of 0.25. The last column contains the **s**urface-**a**veraged **d**onor **a**tom **p**otential **SADAP** = (EP_min_ + EP_max_ + AP)/(ES_pos_ + ES_neg_) as a combined descriptor of steric and electrostatic properties of the potential ligands, which allows an estimation of their reactivity (ES: exposed VdW surface, EP: minimum and maximum potential energy at the VdW surface, AP: averaged potential energy at the VdW surface).

Isocyanide	Exposed VdW Surface, ES (Å^2^)		Extrema for Potential Energies at VdW Surface, EP (kcal/mol)		Average Potential Energies at VdW Surface, AP (kcal/mol)	SADAP
	ES_pos_	ES_neg_	EP_min_	EP_max_	AP_overall_	
CNAr^Tripp2^	0.00	22.23	−38.01	−9.31	−26.65	−3.33
CNAr^Dipp2^	0.00	22.13	−37.64	−8.87	−26.04	−3.28 ^1^
CNPh^i-prop2^	0.00	25.99	−35.47	−5.16	−21.20	−2.38
CNAr^Mes2^	0.00	30.10	−39.20	−7.01	−25.11	−2.37
CNMes	0.00	28.89	−36.79	−6.83	−21.68	−2.26
CN^t^Bu	0.00	31.43	−39.63	−5.86	−22.00	−2.15 ^1^
CN^n^Bu	0.00	31.58	−38.51	−5.52	−21.36	−2.07 ^1^
MIBI	0.02	29.07	−35.96	2.30	−18.65	−1.80
CNH	0.00	31.12	−31.48	−4.62	−16.42	−1.69 ^1^
CNPh*^p^*^-F^	1.67	29.53	−32.02	7.05	−14.10	−1.25 ^1^
CN*p*-FAr^DArF2^	20.50	6.74	−11.49	69.07	16.88	2.73 ^1^

^1^ Value taken from Ref. [11].

**Table 2:** Selected bond lengths (Å) and angles (°) for [Tc(NPh)Cl_3_(PPh_3_)(CN^t^Bu)] (**2a**), [Tc(NPh)Cl_3_(PPh_3_)(CNMes)] (**2b**), [Tc(NPh)Cl_3_(PPh_3_)(CNPh^i-prop2^)] (**2c**), *cis*-[Tc(NPh)Cl_3_(CNAr^Mes2^)_2_] (**3a**) and *trans*-[Tc(NPh)Cl_3_(CNAr^Tripp2^)_2_] (**4b**).

	Tc-N10	Tc-Cl1	Tc-Cl2	Tc-Cl3	Tc-C1	Tc-C2	C1-N1	C2-N2	Tc-N10-C11	N10-Tc-Cl1
**2a**	1.711(2)	2.4131(6)	2.3909(6)	2.4154(6)	2.057(2)		1.133(3)		163.5(2)	160.78(6)
**2b**	1.705(2)	2.3972(7)	2.4033(6)	2.4388(6)	2.037(3)		1.152(3)		164.5(2)	165.24(7)
**2c**	1.725(6)	2.413(2)	2.421(2)	2.409(2)	2.030(8)		1.18(1)		167.4(6)	165.2(2)
**3a**	1.699(3)	2.400(1)	2.391(1)	2.3877(8)	2.056(4)	2.034(3)	1.147(4)	1.160(4)	171.7(3)	166.0(1)
4b *	1.714(5) 1.692(5)	2.347(2) 2.361(2)	2.393(2) 2399(2)	2.395(2) 2.417(2)	2.076(5) 2.095(5)	2.095(5) 2.095(5)	1.150(6) 1.138(6)	1.139(6) 1.135(6)	177.7(2) 178.9(2)	176.9(6) 178.9(2)

* Values for two independent species.

**Table 3:** ν_CN_ Frequencies in the complexes under study compared with the values in the uncoordinated ligands.

	2a (CN^*t*^Bu)	2b (CNMes)	2c (CNPh^i-prop2^)	3a (CNAr^Mes2^)	4a * (CNAr^Dipp2^)	4b (CNAr^Tripp2^)	5 (CN*p*-FAr^DarF2^)	6 (CNPh^pF^)	7 (CNPh^pF^)
	2207	2187	2183	2177	2187	2184	2176	2186	2087
Ligand	2135	2114	2113	2120	2124	2114	2119	2129	2129

* Values taken from Ref. [15].

## Data Availability

Not applicable.

## Supplementary Materials

The following supporting information can be downloaded at: https://www.mdpi.com/article/10.3390/molecules27238546/s1, Table S1: Crystallographic data and data collection parameter; Figure S1: Ellipsoid representation of [Tc(NPh)Cl_3_(PPh_3_)(CN^t^Bu)] ∙ CH_2_Cl_2_ (**2a**). The thermal ellipsoids are set at a 50% probability level. Hydrogen atoms are omitted for clarity. A number of 42 reflections below theta_min_ is missing due to the large unit cell and the data collection with the IPDS T2 measuring routine; Table S2: Selected bond lengths (Å) and angles (°) in [Tc(NPh)Cl_3_(PPh_3_)(CN^t^Bu)] ∙ CH_2_Cl_2_ (**2a**); Figure S2: Ellipsoid representation of [Tc(NPh)Cl_3_(PPh_3_)(CNMes)] (**2b**). The thermal ellipsoids are set at a 50% probability level. Hydrogen atoms are omitted for clarity. A number of 16 reflections below theta_min_ is missing due to the large unit cell and the data collection with the IPDS T2 measuring routine; Table S3: Selected bond lengths (Å) and angles (°) in [Tc(NPh)Cl_3_(PPh_3_)(CNMes)] (**2b**); Figure S3: Ellipsoid representation of [Tc(NPh)Cl_3_(PPh_3_)(CNPh^i-prop2^)] ∙ C_7_H_8_ (**2c**). The thermal ellipsoids are set at a 50% probability level. Hydrogen atoms are omitted for clarity. A number of 11 reflections below theta_min_ is missing due to the large unit cell and the data collection with the IPDS T2 measuring routine. Flack parameter of the non-centrosymmetric structure: 0.06(6); Table S4: Selected bond lengths (Å) and angles (°) in [Tc(NPh)Cl_3_(PPh_3_)(CNPh^i-prop2^)] (**2c**); Figure S4: Ellipsoid representation of *cis*-[Tc(NPh)Cl_3_(CNAr^Mes2^)_2_]∙CH_2_Cl_2_ (**3**). The thermal ellipsoids are set at a 50% probability level. Hydrogen atoms are omitted for clarity. A number of 70 reflections below theta_min_ is missing due to the large unit cell and the data collection with the IPDS T2 measuring routine. Only one molecule of solvent CH_2_Cl_2_ could be located. An overall number of 102 e- has been ‘removed’ from the large. voids, which are contained in the structure, by a solvent mask using OLEX2 (Dolomanov, O.V., Bourhis, L.J., Gildea, R.J, Howard, J.A.K. & Puschmann, H. (2009), J. Appl. Cryst. 42, 339–341). This corresponds to approximately 2.5 more molecules of CH_2_Cl_2_; Table S5: Selected bond lengths (Å) and angles (°) in *cis*-[Tc(NPh)Cl_3_(CNAr^Mes2^)_2_] ∙ CH_2_Cl_2_ (**3**); Figure S5: Ellipsoid representation of *trans*-[Tc(NPh)Cl_3_(CNAr^Tripp2^)_2_] (**4b**). The thermal ellipsoids are set at a 50% probability level. Hydrogen atoms are omitted for clarity. A number of 70 reflections below theta_min_ is missing due to the large unit cell and the data collection with the IPDS T2 measuring routine. One carbon of an isopropyl group has an unusually large ellipsoid due to an elongation of the distal isopropyl moieties. A solvent-accessible void is caused by the molecular packing and does not contain any substantial residual electron density. (b) Visualization of the disorder found for one of the isopropyl groups in the two crystallographically independent species of these large molecules; Table S6: Selected bond lengths (Å) and angles (°) in *trans*-[Tc(NPh)Cl_3_(CNAr^Tripp2^)_2_] (**4b**); Figure S6: Ellipsoid representation of [Tc(NPh)Cl_3_(PPh_3_)(CNp-FAr^DarF2^)] (**5**). The thermal ellipsoids are set at a 50% probability level. Hydrogen atoms are omitted for clarity. The ratio of unique to observed reflections is too low leading to a B alert. This is due to the measurement routine of the IPDS 2T; Table S7: Selected bond lengths (Å) and angles (°) in *trans-*[Tc(NPh)Cl_3_(PPh_3_)(CNp-FAr^DarF2^)] (**5**); Figure S7: ^1^H NMR spectrum of [Tc(NPh)Cl_3_(PPh_3_)(CN^t^Bu)] (**2a**) in CD_2_Cl_2_; Figure S8: IR (KBr) spectrum of p[Tc(NPh)Cl_3_(PPh_3_)(CN^t^Bu)] (**2a**); Figure S9: ^1^H NMR spectrum of [Tc(NPh)(PPh_3_)(CNMes)Cl_3_] (**2b**) in CD_2_Cl_2_; Figure S10: ^1^H,^1^H COSY NMR spectrum of *cis*-[Tc(NPh)(PPh_3_)(CNMes)Cl_3_] (**2b**) in CD_2_Cl_2_; Figure S11: IR (KBr) spectrum of [Tc(NPh)(PPh_3_)(CNMes)Cl_3_] (**2b**); Figure S12: ^1^H NMR spectrum of [Tc(NPh)Cl_3_(PPh_3_)(CNPh^i-prop2^)] (**2c**) in CD_2_Cl_2_; Figure S13: IR (KBr) spectrum of [Tc(NPh)Cl_3_(PPh_3_)(CNPh^i-prop2^)] (**2c**); Figure S14: ^1^H NMR spectrum of isomers of [Tc(NPh)Cl_3_(PPh_3_)(CNAr^Mes2^)] (**3a**) in CD_2_Cl_2_; Figure S15: ^1^H NMR spectrum of trans-[Tc(NPh)Cl_3_(CNAr^Tripp2^)_2_] (**4b**) in CD_2_Cl_2_; Figure S16: IR (KBr) spectrum of *trans*-[Tc(NPh)(CNAr^Tripp2^)Cl_3_] (**4b**); Figure S17: ^1^H NMR spectrum of [Tc(NPh)Cl_3_(PPh_3_)(CNp-FAr^DarF2^)] (**5**) in CD_2_Cl_2_; Figure S18: ^19^F NMR spectrum of [Tc(NPh)Cl_3_(PPh_3_)(CNp-FAr^DarF2^)] (**5**) in CD_2_Cl_2_; Figure S19: ^19^F NMR spectra recorded during the reaction of [Tc(NPh)Cl_3_(PPh_3_)(CNp-FAr^DarF2^)] (**5**) with CNp-FAr^DarF2^ in THF. Spectrum 1 was recorded after the addition of one equivalent of CNp-FAr^DarF2^ to **5**. Spectrum 2 was recorded after heating the previously obtained solution for 10 min in boiling THF. Spectrum 3 was recorded after heating a solution of complex **5** for 10 min in boiling THF; Figure S20: IR (KBr) spectrum of [Tc(NPh)Cl_3_(PPh_3_)(CNp-FAr^DarF2^)] (**5**); Figure S21: ^1^H NMR spectrum of [Tc(NPh)Cl_3_(PPh_3_)(CNPh^pF^)] (**6**) in CD_2_Cl_2_; Figure S22: ^19^F NMR spectrum of [Tc(NPh)Cl_3_(PPh_3_)(CNPh^pF^)] (**6**) in CD_2_Cl_2_; Figure S23: ^31^P{^1^H} NMR spectrum of [Tc(NPh)Cl_3_(PPh_3_)(CNPh^pF^)] (**6**) in CD_2_Cl_2_; Figure S24: IR (KBr) spectrum of [Tc(NPh)Cl_3_(PPh_3_)(CNPh^pF^)] (**6**); Figure S25: ^1^H NMR spectrum of [Tc(CNPh^*p*-F^)_6_][PF_6_] (**7**) in CD_2_Cl_2_; Figure S26: ^19^F NMR spectrum of [Tc(CNPh^*p*-F^)_6_][PF_6_] (**7**) in CD_2_Cl_2_; Figure S27: ^99^Tc NMR spectrum of [Tc(CNPh^pF^)_6_][PF_6_] (**7**) in CD_2_Cl_2_; Figure S28: IR (KBr) spectrum of [Tc(CNPh^*p*-F^)_6_][PF_6_] (**7**); Table S8: Calculated electrostatic potential surface properties of the isocyanide carbon atom at the Van der Waals (VdW) boundary for structures optimized at the B3LYP/6-311++G** level. Surface properties were evaluated at ρ = 0.001 level using an electrostatic potential map basis with a grid-point spacing of 0.25. The last column contains the Surface-Averaged Donor Atom Potential SADAP = (EP_min_ + EP_max_ + AP)/(ES_pos_ + ES_neg_) as a combined descriptor of steric and electrostatic properties of the potential ligands, which allows an estimation of their reactivity; Table S9: LP_c_/π*_CN_ properties of some representative, free isocyanides, number of donated σ_CTc_ electrons (#e^−^; LP_c_→LP*_Tc_), and second order perturbation parameters (interaction energy *E*; energy difference between the two orbitals *E*_i_-*E*_j_; overlap parameter *A*) for LP_Tc_→π*_CN_ in the model complexes [TcO_3_(CO/CNR)]^+^ and [Tc(CO)_5_(CO/CNR)]^+^ (R = Ph^F5^, Ph*^p-^*^F^, Ph, *^t^*Bu, Ar^DArF2^).
