# Supplementary material for: The Chemistry of Phenylimidotechnetium(V) Complexes with Isocyanides: Steric and Electronic Factors
Source: Molecules. 2022 Dec 4;27(23):8546. doi: 10.3390/molecules27238546 (PMC9736260; doi:10.3390/molecules27238546)
Supplement: Supplementary file 1 [file molecules-27-08546-s001.zip › molecules-2028667-supplementary.pdf]

Supporting information for the paper entitled:

The Chemistry of Phenylimidotechnetium(V) Complexes with Isocyanides: Steric and Electronic Factors

Guilhem Claude,[1] Laura Zeh,[1] Maximilian Roca Jungfer,[1] Adelheid Hagenbach,[1] Joshua R. Figueroa[2], and Ulrich Abram[1*]*

[1]Freie Universität Berlin, Institute of Chemistry and Biochemistry, Fabeckstr. 34–36, 14195 Berlin, Germany

E-mail: ulrich.abram@fu-berlin.de

[2] Department of Chemistry and Biochemistry, University of California, San Diego, La Jolla, California 92093, United States

E-mail: jsfig@ucsd.edu

Table content

Crystallographic data 5

Table S1: Crystallographic data and data collection parameters 5

Figure S1: Ellipsoid representation of *fac*-[Tc(NPh)Cl$_3$(PPh$_3$)(CN^{t}Bu)] · CH$_2$Cl$_2$ (**2a**). The thermal ellipsoids are set at a 50% probability level. Hydrogen atoms are omitted for clarity. A number of 42 reflections below theta$_{min}$ is missing due to the large unit cell and the data collection with the IPDS T2 measuring routine. 7

Table S2: Selected bond lengths (Å) and angles (°) in *fac*- [Tc(NPh)Cl$_3$(PPh$_3$)(CN^{t}Bu)] · CH$_2$Cl$_2$ (**2a).** 7

Figure S2. Ellipsoid representation of *fac*-[Tc(NPh)Cl$_3$(PPh$_3$)(CNMes)] (**2b**). The thermal ellipsoids are set at a 50% probability level. Hydrogen atoms are omitted for clarity. A number of 16 reflections below theta$_{min}$ is missing due to the large unit cell and the data collection with the IPDS T2 measuring routine. 8

Table S3: Selected bond lengths (Å) and angles (°) in *fac*- [Tc(NPh)Cl$_3$(PPh$_3$)(CNMes)] (**2b).** 8

Figure S3: Ellipsoid representation of *fac*-[Tc(NPh)Cl$_3$(PPh$_3$)(CNPh$^{i\text{-}prop2}$)] · C$_7$H$_8$ (**2c**). The thermal ellipsoids are set at a 50% probability level. Hydrogen atoms are omitted for clarity. A number of 11 reflections below theta$_{min}$ is missing due to the large unit cell and the data collection with the IPDS T2 measuring routine. Flack parameter of the non-centrosymmetric structure: 0.06(6). 9

Table S4: Selected bond lengths (Å) and angles (°) in *fac*-[Tc(NPh)Cl$_3$(PPh$_3$)(CNPh$^{i\text{-}prop2}$)] (**2c**). 9

Figure S4: Ellipsoid representation of *cis,fac*-[Tc(NPh)Cl$_3$(CNAr^{Mes2})$_2$]·CH$_2$Cl$_2$ (**3**). The thermal ellipsoids are set at a 50% probability level. Hydrogen atoms are omitted for clarity. A number of 70 reflections below theta$_{min}$ is missing due to the large unit cell and the data collection with the IPDS T2 measuring routine. Only one molecule of solvent CH$_2$Cl$_2$ could be located. An overall number of 102 e- has been 'removed' from the large. Voids, which are contained in the structure, by a solvent mask using OLEX2 (Dolomanov, O.V., Bourhis, L.J., Gildea, R.J, Howard, J.A.K. & Puschmann, H. (2009), J. Appl. Cryst. 42, 339-341). This corresponds to approximately 2.5 more molecules of CH$_2$Cl$_2$. 10

Table S5: Selected bond lengths (Å) and angles (°) in *cis, fac*-[Tc(NPh)Cl$_3$CNAr^{Mes2})$_2$] · CH$_2$Cl$_2$ (**3**). 10

Figure S5: a) Ellipsoid representation of *trans,mer*-[Tc(NPh)Cl$_3$(CNAr^{Tripp2})$_2$] (**4b**). The thermal ellipsoids are set at a 50% probability level. Hydrogen atoms are omitted for clarity. A number of 70 reflections below theta$_{min}$ is missing due to the large unit cell and the data collection with the IPDS T2 measuring routine. One carbon of an isopropyl group has an unusually large ellipsoid due to an elongation of the distal isopropyl moieties. A solvent accessible void is caused by the molecular packing and does not contain any substantial residual electron density. b) Visualization of the disorder found for one of the isopropyl groups in the two crystallographically independent species of these large molecules. 11

Table S6: Selected bond lengths (Å) and angles (°) in *trans,mer*-[Tc(NPh)Cl$_3$CNAr^{Tripp2})$_2$] (**4b**). 11

Figure S6: Ellipsoid representation of *fac*-[Tc(NPh)Cl_3(PPh_3)(CN*p*-FAr^{DarF2})] (**5**). The thermal ellipsoids are set at a 50% probability level. Hydrogen atoms are omitted for clarity. The ratio of unique to observed reflections is too low leading to a B alert. This is due to the measurement routine of the IPDS 2T. 12

Table S7: Selected bond lengths (Å) and angles (°) in *fac*-[Tc(NPh)Cl_3(PPh_3)(CN*p*-FAr^{DarF2})] (**5**). 12

Spectroscopic data 13

Figure S7: ^{1}H NMR spectrum of *fac*-[Tc(NPh)Cl_3(PPh_3)(CN^tBu)] (**2a**) in CD_2Cl_2. 13

Figure S8: IR (KBr) spectrum of *fac*-[Tc(NPh)Cl_3(PPh_3)(CN^tBu)] (**2a**) 13

Figure S9: ^{1}H NMR spectrum of 14

Figure S10: ^{1}H,^{1}H COSY NMR spectrum of *fac*-[Tc(NPh)(PPh_3)(CNMes)Cl_3] (**2b**) in CD_2Cl_2 14

Figure S11: IR (KBr) spectrum of *fac*-[Tc(NPh)(PPh_3)(CNMes)Cl_3] (**2b**). 15

Figure S12: ^{1}H NMR spectrum of *fac*-[Tc(NPh)Cl_3(PPh_3)($CNPh^{i\text{-}prop2}$)] (**2c**) in CD_2Cl_2. 15

Figure S13: IR (KBr) spectrum of *fac*-[Tc(NPh)Cl_3(PPh_3)($CNPh^{i\text{-}prop2}$)] (**2c**). 16

Figure S14: ^{1}H NMR spectrum of isomers of *fac*-[Tc(NPh)Cl_3(PPh_3)($CNAr^{Mes2}$)] (**3a**) in CD_2Cl_2. 16

Figure S15: ^{1}H NMR spectrum of *trans,mer*-[Tc(NPh)Cl_3($CNAr^{Tripp2}$)$_2$] (**4b**) in CD_2Cl_2. 17

Figure S16: IR (KBr) spectrum of *trans,mer*-[Tc(NPh)($CNAr^{Tripp2}$)Cl_3] (**4b**). 17

Figure S17: ^{1}H NMR spectrum of *fac*-[Tc(NPh)Cl_3(PPh_3)(CN*p*-FAr^{DarF2})] (**5**) in CD_2Cl_2. 18

Figure S18: ^{19}F NMR spectrum of *fac*- [Tc(NPh)Cl_3(PPh_3)(CN*p*-FAr^{DarF2})] (**5**) in CD_2Cl_2. 18

Figure S19: ^{19}F NMR spectra recorded during the reaction of *fac*-[Tc(NPh)Cl_3(PPh_3)(CN*p*-FAr^{DarF2})] (**5**) with CN*p*-FAr^{DarF2} in THF. Spectrum 1 was recorded after the addition of one equivalent of CN*p*-FAr^{DarF2} to **5**. Spectrum 2 was recorded after heating the previously obtained solution for 10 min in boiling THF. Spectrum 3 was recorded after heating a solution of complex **5** for 10 min in boiling THF. 19

Figure S20: IR (KBr) spectrum of *fac*-[Tc(NPh)Cl_3(PPh_3)(CN*p*-FAr^{DarF2})] (**5**). 19

Figure S21: ^{1}H NMR spectrum of *fac*-[Tc(NPh)Cl_3(PPh_3)($CNPh^{pF}$)] (**6**) in CD_2Cl_2. 20

Figure S22: ^{19}F NMR spectrum of *fac*-[Tc(NPh)Cl_3(PPh_3)($CNPh^{pF}$)] (**6**) in CD_2Cl_2. 20

Figure S23: ^{31}P{^{1}H} NMR spectrum of *fac*-[Tc(NPh)Cl_3(PPh_3)($CNPh^{pF}$)] (**6**) in CD_2Cl_2 21

Figure S24: IR (KBr) spectrum of *fac*-[Tc(NPh)Cl_3(PPh_3)($CNPh^{pF}$)] (**6**). 21

Figure S25: ^{1}H NMR spectrum of *fac*-[Tc($CNPh^{pF}$)$_6$][PF_6] (**7**) in CD_2Cl_2. 22

Figure S26: ^{19}F NMR spectrum of [Tc($CNPh^{pF}$)$_6$][PF_6] (**7**) in CD_2Cl_2. 22

Figure S27: ^{99}Tc NMR spectrum of [Tc($CNPh^{pF}$)$_6$][PF_6] (**7**) in CD_2Cl_2. 23

Figure S28: IR (KBr) spectrum of [Tc($CNPh^{pF}$)$_6$][PF_6] (**7**). 23

DFT Calculations 24

Table S8. Calculated electrostatic potential surface properties of the isocyanide carbon atom at the Van der Waals (VdW) boundary for structures optimized at the B3LYP/6-311++G** level. Surface properties were evaluated at ρ = 0.001 level using an

electrostatic potential map basis with a grid-point spacing of 0.25. The last column contains the Surface-Averaged Donor Atom Potential SADAP = (EP_{min} + EP_{max} + AP)/(ES_{pos} + ES_{neg}) as a combined descriptor of steric and electrostatic properties of the potential ligands, which allows an estimation of their reactivity. 24

Table S9. LP_c/π^*_{CN} properties of some representative, free isocyanides, number of donated σ_{CTc} electrons (#e^-; $LP_c \rightarrow LP^*_{Tc}$) and second order perturbation parameters (interaction energy *E*; energy difference between the two orbitals E_i-E_j; overlap parameter *A*) for $LP_{Tc} \rightarrow \pi^*_{CN}$ in the model complexes $[TcO_3(CO/CNR)]^+$ and $[Tc(CO)_5(CO/CNR)]^+$ (R = Ph^{F5}, $Ph^{p\text{-}F}$, Ph, ^{t}Bu, Ar^{DArF2})... 28

Crystallographic data

The intensities for the X–ray determinations were collected on STOE IPDS 2T with Mo Kα radiation. The space groups were determined by the detection of systematical absences. Absorption corrections were carried out by integration methods (Coppens, P. The Evaluation of Absorption and Extinction in Single-Crystal Structure Analysis. Crystallographic Computing; Copenhagen, Muksgaard, **1979)**. Structure solution and refinement were performed with the SHELX program package. Hydrogen atoms were derived from the final Fourier maps and refined or placed at calculated positions and treated with the ‘riding model’ option of SHELXL. The representation of molecular structures was done using the program DIAMOND 4.2.2. Additional information on the structure determinations has been deposited with the Cambridge Crystallographic Data Centre.

Table S1: Crystallographic data and data collection parameters

	fac-[Tc(NPh)Cl_3(PPh_3)(CN^tBu)] · CH_2Cl_2 (**2a**)	*fac*-[Tc(NPh)Cl_3(PPh_3)-(CNMes)] (**2b**)	*fac*-[Tc(NPh)Cl_3(PPh_3)-($CNPh^{i\text{-}prop2}$)] · C_7H_8 (**2c**)
Empirical formula	$C_{30}H_{31}Cl_5N_2PTc$	$C_{34}H_{31}Cl_3N_2PTc$	$C_{44}H_{45}Cl_3N_2PTc$
Formula weight	725.79	702.93	837.14
Temperature/K	200.0	200.0	293(2)
Crystal system	triclinic	monoclinic	orthorhombic
Space group	P-1	$P2_1/n$	$Pca2_1$
a/Å	9.8713(7)	9.5714(7)	21.988(4)
b/Å	12.5882(9)	20.816(1)	10.556(2)
c/Å	14.1290(1)	16.296(1)	17.683(4)
α/°	80.628(6)	90	90
β/°	74.909(6)	99.504(5)	90
γ/°	73.409(6)	90	90
Volume/Å^3	1617.1(2)	3202.2(3)	4104.1(14)
Z	2	4	4
ρ_{calc}g / cm^3	1.491	1.458	1.355
μ / mm^{-1}	0.930	0.776	0.618
F(000)	736.0	1432.0	1728.0
Crystal size / mm^3	0.66 × 0.56 × 0.38	0.3 × 0.24 × 0.2	0.35 × 0.2 × 0.2
Radiation	MoKα (λ = 0.71073)	MoKα (λ = 0.71073)	MoKα (λ = 0.71073)
2Θ range for data collection/°	9.484 to 51.992	7.242 to 58.496	6.768 to 58.482
Index ranges	-10 ≤ h ≤ 12, -15 ≤ k ≤ 15, -17 ≤ l ≤ 15	-10 ≤ h ≤ 13, -28 ≤ k ≤ 28, -22 ≤ l ≤ 22	-19 ≤ h ≤ 30, -11 ≤ k ≤ 14, -23 ≤ l ≤ 24
Reflections collected	13325	25489	16632
Independent reflections	6302 [R_{int} = 0.0473, R_{sigma} = 0.0355]	8612 [R_{int} = 0.0927, R_{sigma} = 0.0795]	9691 [R_{int} = 0.1047, R_{sigma} = 0.1771]
Data/restraints/parameters	6302/0/355	8612/0/373	9691/521/465
Goodness-of-fit on F^2	1.045	0.887	0.802
Final R indexes [I>=2σ (I)]	R_1 = 0.0303, wR_2 = 0.0769	R_1 = 0.0377, wR_2 = 0.0846	R_1 = 0.0552, wR_2 = 0.0903
Final R indexes [all data]	R_1 = 0.0346, wR_2 = 0.0785	R_1 = 0.0599, wR_2 = 0.0903	R_1 = 0.1262, wR_2 = 0.1065
Largest diff. peak/hole / e Å^{-3}	0.50/-0.94	0.75/-1.34	0.48/-0.50
Flack parameter	-	-	-0.06(6)
CCDC access code	2208603	2208604	2208605

Table S1 (continued): Crystallographic data and data collection parameters.

	cis-[Tc(NPh)Cl_3($CNAr^{Mes2}$)$_2$] · CH_2Cl_2 (**3**)	*trans*-[Tc(NPh)Cl_3($CNAr^{Tripp2}$)$_2$] (**4b**)	*fac*-[Tc(NPh)Cl_3(PPh_3)-(CNp-FAr^{DarF2})] (**5**)
Empirical formula	$C_{57}H_{57}Cl_5N_3Tc$	$C_{80}H_{103}Cl_3N_3Tc$	$C_{47}H_{28}Cl_3F_{13}N_2PTc$
Formula weight	1059.30	1311.00	1103.03
Temperature/K	200.0	200.0	200.0
Crystal system	triclinic	monoclinic	monoclinic
Space group	P-1	$P2_1/n$	$P2_1/n$
a/Å	12.4573(9)	24.4011(9)	8.5399(8)
b/Å	13.3922(11)	24.9304(7)	22.855(2)
c/Å	19.8431(16)	28.0596(13)	23.501(2)
α/°	96.377(7)	90	90
β/°	92.599(6)	108.814(3)	98.416(7)
γ/°	114.514(6)	90	90
Volume / $Å^3$	2978.1(4)	16157.5(11)	4537.4(7)
Z	2	8	4
ρ_{calc} g/cm^3	1.181	1.078	1.615
μ / mm^{-1}	0.501	0.317	0.620
F(000)	1096.0	5584.0	2200.0
Crystal size / mm^3	0.6 × 0.36 × 0.2	0.9 × 0.72 × 0.46	0.2 × 0.133 × 0.1
Radiation	MoKα (λ = 0.71073)	MoKα (λ = 0.71073)	MoKα (λ = 0.71073)
2Θ range for data collection/°	6.698 to 51.996	6.646 to 49.998	3.932 to 51
Index ranges	-15 ≤ h ≤ 15, -13 ≤ k ≤ 16, -24 ≤ l ≤ 24	-29 ≤ h ≤ 29, -29 ≤ k ≤ 29, -33 ≤ l ≤ 24	-10 ≤ h ≤ 10, -27 ≤ k ≤ 27, -27 ≤ l ≤ 28
Reflections collected	25752	82662	27078
Independent reflections	11656 [R_{int} = 0.0749, R_{sigma} = 0.1151]	28300 [R_{int} = 0.1080, R_{sigma} = 0.0972]	8443 [R_{int} = 0.1506, R_{sigma} = 0.2363]
Data/restraints/parameters	11656/0/607	28300/1886/1498	8443/854/621
Goodness-of-fit on F^2	0.833	0.999	0.669
Final R indexes [I>=2σ (I)]	R_1 = 0.0471, wR_2 = 0.0832	R_1 = 0.0817, wR2 = 0.2151	R_1 = 0.0477, wR_2 = 0.0657
Final R indexes [all data]	R_1 = 0.0915, wR_2 = 0.0929	R_1 = 0.1273, wR2 = 0.2422	R_1 = 0.1479, wR_2 = 0.0819
Largest diff. peak/hole / e $Å^{-3}$	0.41/-0.58	1.59/-1.00	0.70/-0.70
CCDC access code	2208606	2208607	2208608

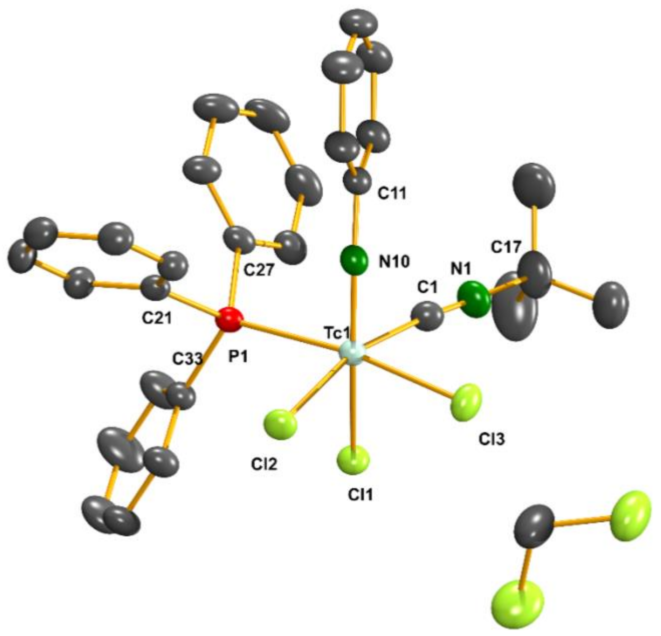

Figure S1: Ellipsoid representation of *fac*-[Tc(NPh)Cl_3(PPh_3)(CN^tBu)] · CH_2Cl_2 (**2a**). The thermal ellipsoids are set at a 50% probability level. Hydrogen atoms are omitted for clarity. A number of 42 reflections below $theta_{min}$ is missing due to the large unit cell and the data collection with the IPDS T2 measuring routine.

Table S2: Selected bond lengths (Å) and angles (°) in *fac*- [Tc(NPh)Cl_3(PPh_3)-(CN^tBu)] · CH_2Cl_2 (**2a).**

Tc1-Cl2	2.3909(6)	P1-C27	1.824(2)	Tc1-Cl3	2.4154(6)
Tc1-Cl1	2.4131(6)	P1-C33	1.819(2)	Tc1-N10	1.7114(18)
Tc1-P1	2.4602(6)	P1-C21	1.828(2)	Tc1-C1	2.057(2)
N10-C11	1.388(3)	C1-N1	1.133(3)	N1-C17	1.470(3)
Cl2-Tc1-Cl1	91.06(22)	Cl1-Tc1-P1	84.90(2)	C11-N10-Tc1	163.55(2)
Cl2-Tc1-P1	87.21(2)	Cl1-Tc1-Cl3	89.85(2)	C1-Tc1-P1	99.94(7)
Cl2-Tc1-Cl3	86.79(2)	Cl3-Tc1-P1	171.95(2)	N1-C1-Tc1	173.1(2)
C1-Tc1-Cl2	165.95(7)	C1-Tc1-Cl1	77.68(6)	C1-N1-C17	178.0(3)

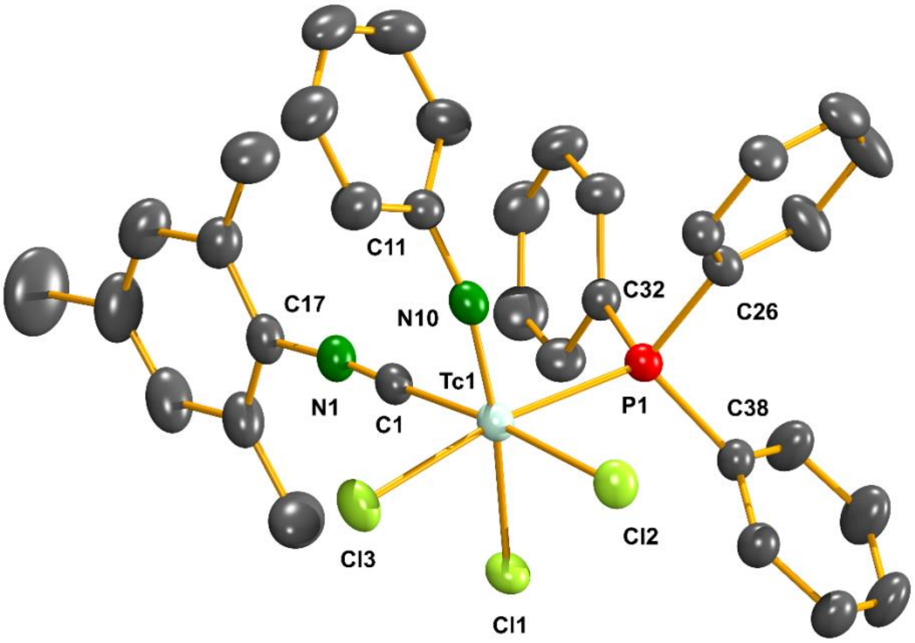

Figure S2. Ellipsoid representation of *fac*-[Tc(NPh)Cl_3(PPh_3)(CNMes)] (**2b**). The thermal ellipsoids are set at a 50% probability level. Hydrogen atoms are omitted for clarity. A number of 16 reflections below $theta_{min}$ is missing due to the large unit cell and the data collection with the IPDS T2 measuring routine.

Table S3: Selected bond lengths (Å) and angles (°) in *fac*- [Tc(NPh)Cl_3(PPh_3)-(CNMes)] (**2b).**

Tc1-Cl2	2.4033(6)	P1-C26	1.827(3)	Tc1-Cl3	2.4388(6)
Tc1-Cl1	2.3972(7)	P1-C32	1.820(2)	Tc1-N10	1.705(22)
Tc1-P1	2.4654(6)	P1-C26	1.827(3)	Tc1-C1	2.037(3)
N10-C11	1.382(3)	C1-N1	1.152(3)	N1-C17	1.403(3)
Cl2-Tc1-Cl1	93.88(3)	Cl1-Tc1-P1	84.00(2)	C11-N10-Tc1	164.51(2)
Cl2-Tc1-P1	88.00(2)	Cl2-Tc1-Cl3	88.31(2)	C1-Tc1-P1	92.33(7)
Cl1-Tc1-Cl3	88.78(2)	Cl3-Tc1-P1	171.65(2)	N1-C1-Tc1	177.3(2)
C1-Tc1-Cl2	174.06(7)	C1-Tc1-Cl1	80.26(7)	C1-N1-C17	173.7(3)

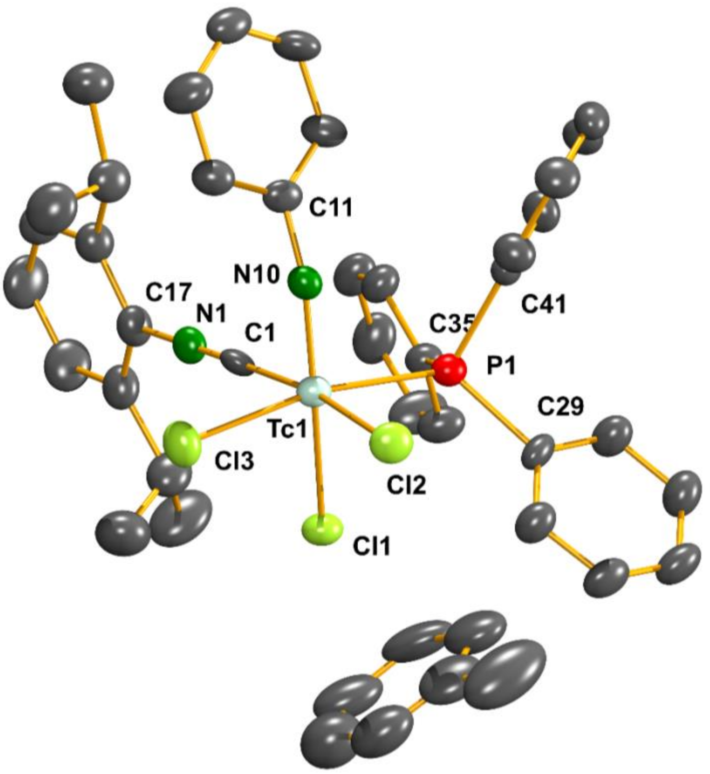

Figure S3: Ellipsoid representation of *fac*-[Tc(NPh)Cl_3(PPh_3)($CNPh^{i\text{-}prop2}$)] · C_7H_8 (**2c**). The thermal ellipsoids are set at a 50% probability level. Hydrogen atoms are omitted for clarity. A number of 11 reflections below $theta_{min}$ is missing due to the large unit cell and the data collection with the IPDS T2 measuring routine. Flack parameter of the non-centrosymmetric structure: 0.06(6).

Table S4: Selected bond lengths (Å) and angles (°) in *fac*-[Tc(NPh)Cl_3(PPh_3)($CNPh^{i\text{-}prop2}$)] (**2c**).

Tc1-Cl2	2.421(2)	P1-C41	1.834(9)	Tc1-Cl3	2.409(2)
Tc1-Cl1	2.413(2)	P1-C35	1.808(1)	Tc1-N10	1.725(6)
Tc1-P1	2.452(2)	P1-C29	1.854(9)	Tc1-C1	2.030(8)
N10-C11	1.374(1)	C1-N1	1.1834(1)	N1-C17	1.390(1)
Cl2-Tc1-Cl1	94.62(8)	Cl1-Tc1-P1	79.67(8)	C11-N10-Tc1	167.4(6)
Cl2-Tc1-P1	92.96(1)	Cl2-Tc1-Cl3	87.35(1)	C1-Tc1-P1	92.7(3)
Cl1-Tc1-Cl3	87.35(1)	Cl3-Tc1-P1	87.75(9)	N1-C1-Tc1	176.7(9)
C1-Tc1-Cl2	172.0(3)	C1-Tc1-Cl1	80.9(2)	C1-N1-C17	178.8(9)

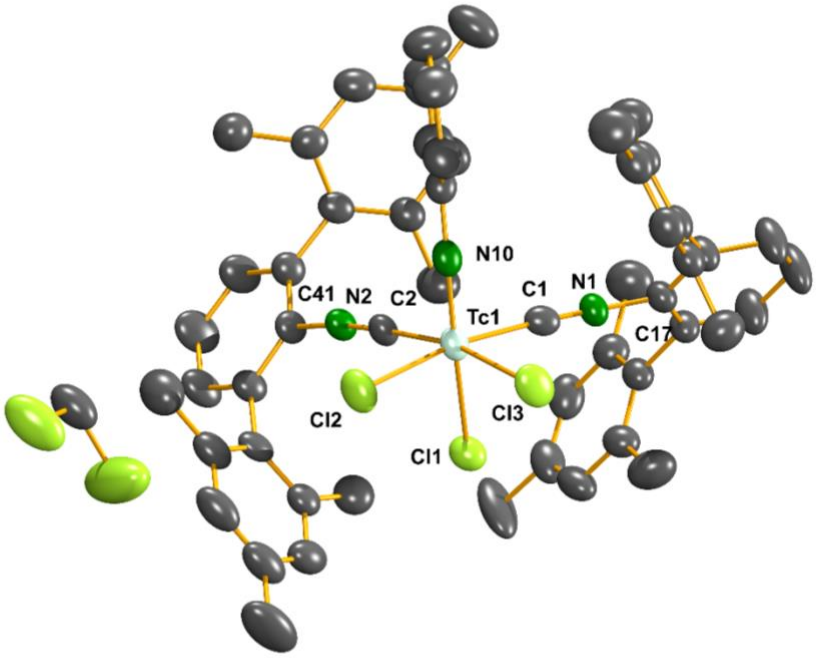

Figure S4: Ellipsoid representation of *cis,fac*-[Tc(NPh)Cl_3($CNAr^{Mes2}$)$_2$]·CH_2Cl_2 (**3**). The thermal ellipsoids are set at a 50% probability level. Hydrogen atoms are omitted for clarity. A number of 70 reflections below $theta_{min}$ is missing due to the large unit cell and the data collection with the IPDS T2 measuring routine. Only one molecule of solvent CH_2Cl_2 could be located. An overall number of 102 e- has been 'removed' from the large. Voids, which are contained in the structure, by a solvent mask using OLEX2 (Dolomanov, O.V., Bourhis, L.J., Gildea, R.J, Howard, J.A.K. & Puschmann, H. (2009), J. Appl. Cryst. 42, 339-341). This corresponds to approximately 2.5 more molecules of CH_2Cl_2.

Table S5: Selected bond lengths (Å) and angles (°) in *cis, fac*-[Tc(NPh)Cl_3-$CNAr^{Mes2}$)$_2$] · CH_2Cl_2 (**3**).

Tc1-Cl2	2.3909(1)	N1-C17	1.392(5)	Tc1-Cl3	2.3877(9)
Tc1-Cl1	2.4004(1)	N2-C41	1.1411(4)	Tc1-N10	1.699(3)
C2-N2	1.160(4)	Tc1-C2	2.034(3)	Tc1-C1	2.056(4)
N10-C11	1.380(4)	C1-N1	1.147(4)		
Cl2-Tc1-Cl1	87.70(9)	Cl1-Tc1-C2	86.41(2)	C11-N10-Tc1	176.9(6)
Cl2-Tc1-C2	92.21(1)	Cl2-Tc1-Cl3	174.04(9)	C1-Tc1-C2	171.7(2)
Cl1-Tc1-Cl3	86.40(9)	N2-C2-Tc1	175.4(4)	N1-C1-Tc1	177.9(5)
C1-Tc1-Cl2	88.65(1)	C1-Tc1-Cl1	85.38(2)	C1-N1-C31	179.3(6)

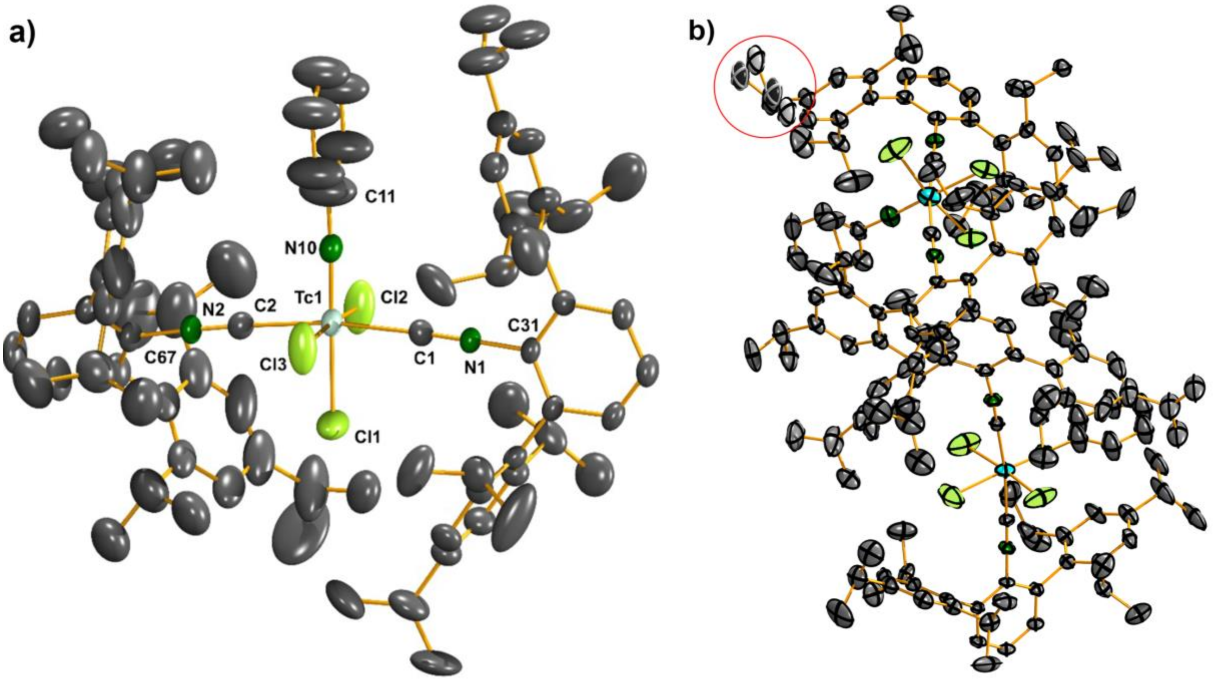

Figure S5: a) Ellipsoid representation of *trans,mer*-[Tc(NPh)Cl_3(CNAr^{Tripp2})$_2$] (**4b**). The thermal ellipsoids are set at a 50% probability level. Hydrogen atoms are omitted for clarity. A number of 70 reflections below theta$_{min}$ is missing due to the large unit cell and the data collection with the IPDS T2 measuring routine. One carbon of an isopropyl group has an unusually large ellipsoid due to an elongation of the distal isopropyl moieties. A solvent accessible void is caused by the molecular packing and does not contain any substantial residual electron density. b) Visualization of the disorder found for one of the isopropyl groups in the two crystallographically independent species of these large molecules.

Table S6: Selected bond lengths (Å) and angles (°) in *trans,mer*-[Tc(NPh)Cl_3-CNAr^{Tripp2})$_2$] (**4b**).

Tc1-Cl2	2.3933(2)	N1-C1	1.150(6)	Tc1-Cl3	2.3946(2)
Tc1-Cl1	2.3470(2)	N2-C67	1.1416(6)	Tc1-N10	1.714(5)
C2-N2	1.139(6)	Tc1-C2	2.097(5)	Tc1-C1	2.076(5)
N10-C11	1.361(1)	N1-C31	1.409(6)		
Cl2-Tc1-Cl1	87.70(9)	Cl1-Tc1-C2	86.41(2)	C11-N10-Tc1	176.9(6)
Cl2-Tc1-C2	92.21(1)	Cl2-Tc1-Cl3	174.04(9)	C1-Tc1-C2	171.7(2)
Cl1-Tc1-Cl3	86.40(9)	N2-C2-Tc1	175.4(4)	N1-C1-Tc1	177.9(5)
C1-Tc1-Cl2	91.69(1)	C1-Tc1-Cl1	85.38(2)	C1-N1-C31	179.3(6)

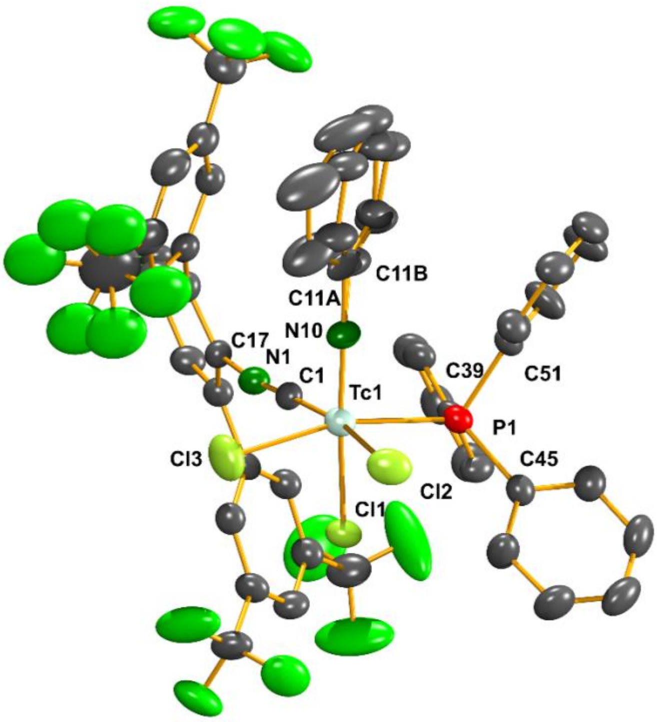

Figure S6: Ellipsoid representation of *fac*-[Tc(NPh)Cl_3(PPh_3)(CN*p*-FAr^{DarF2})] (**5**). The thermal ellipsoids are set at a 50% probability level. Hydrogen atoms are omitted for clarity. The ratio of unique to observed reflections is too low leading to a B alert. This is due to the measurement routine of the IPDS 2T.

Table S7: Selected bond lengths (Å) and angles (°) in *fac*-[Tc(NPh)Cl_3(PPh_3)(CN*p*-FAr^{DarF2})] (**5**).

Tc1-Cl2	2.4338(2)	P1-C45	1.821(5)	Tc1-Cl3	2.3890(2)
Tc1-Cl1	2.3977(2)	P1-C39	1.793(5)	Tc1-N10	1.710(4)
Tc1-P1	2.4423(2)	P1-C51	1.812(5)	Tc1-C1	2.009(5)
N10-C11A	1.395(1)	C1-N1	1.176(6)	N1-C17	1.401(6)
Cl2-Tc1-Cl1	96.17(5)	Cl1-Tc1-P1	90.42(5)	C11A-N10-Tc1	168.3(1)
Cl2-Tc1-P1	77.65(5)	Cl2-Tc1-Cl3	86.94(6)	C1-Tc1-P1	94.83(2)
Cl1-Tc1-Cl3	87.80(6)	Cl3-Tc1-P1	164.21(6)	N1-C1-Tc1	174.9(5)
C1-Tc1-Cl2	78.68(2)	C1-Tc1-Cl1	171.66(2)	C1-N1-C17	172.0(5)

Spectroscopic data

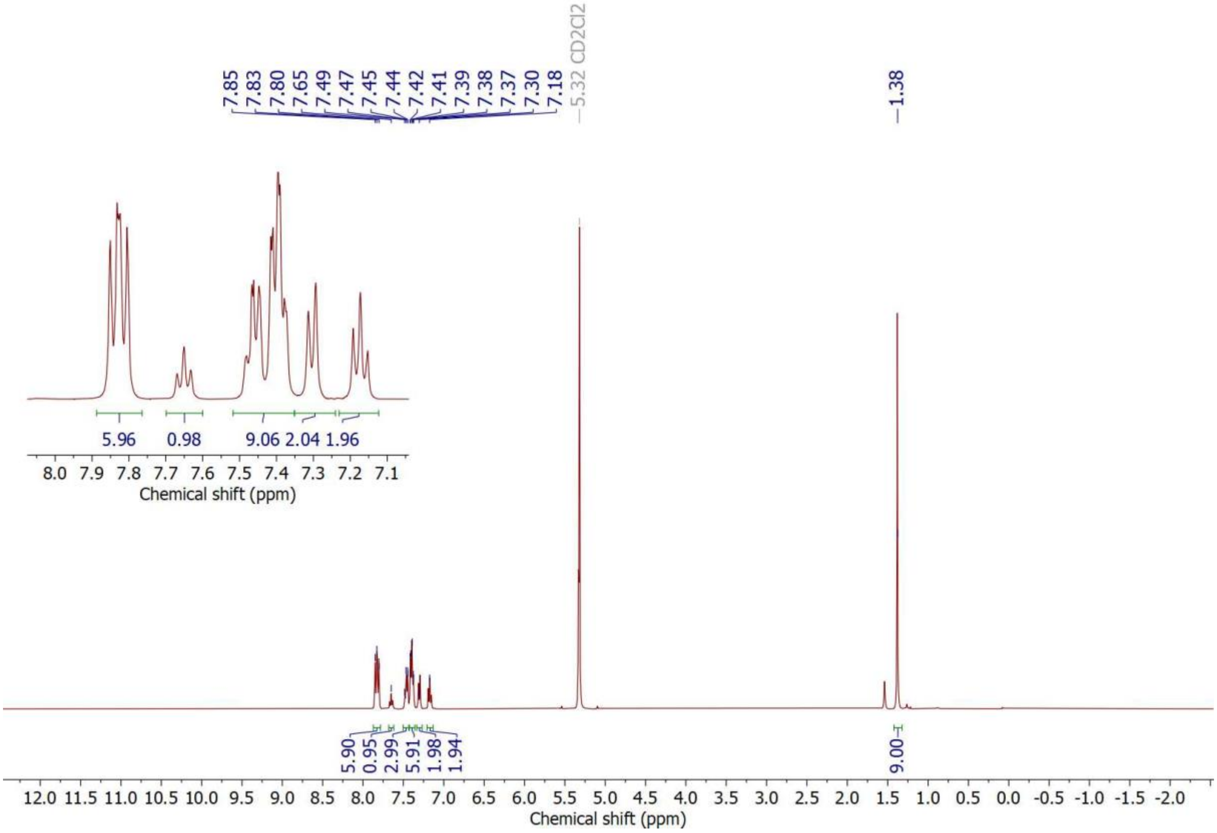

Figure S7: ^{1}H NMR spectrum of *fac*-[Tc(NPh)Cl_3(PPh_3)(CN^tBu)] (**2a**) in CD_2Cl_2.

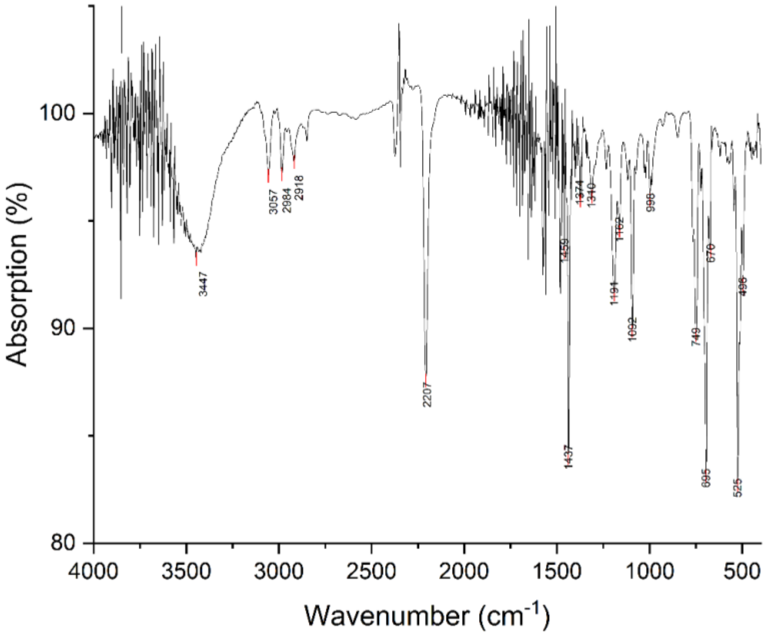

Figure S8: IR (KBr) spectrum of *fac*-[Tc(NPh)Cl_3(PPh_3)(CN^tBu)] (**2a**).

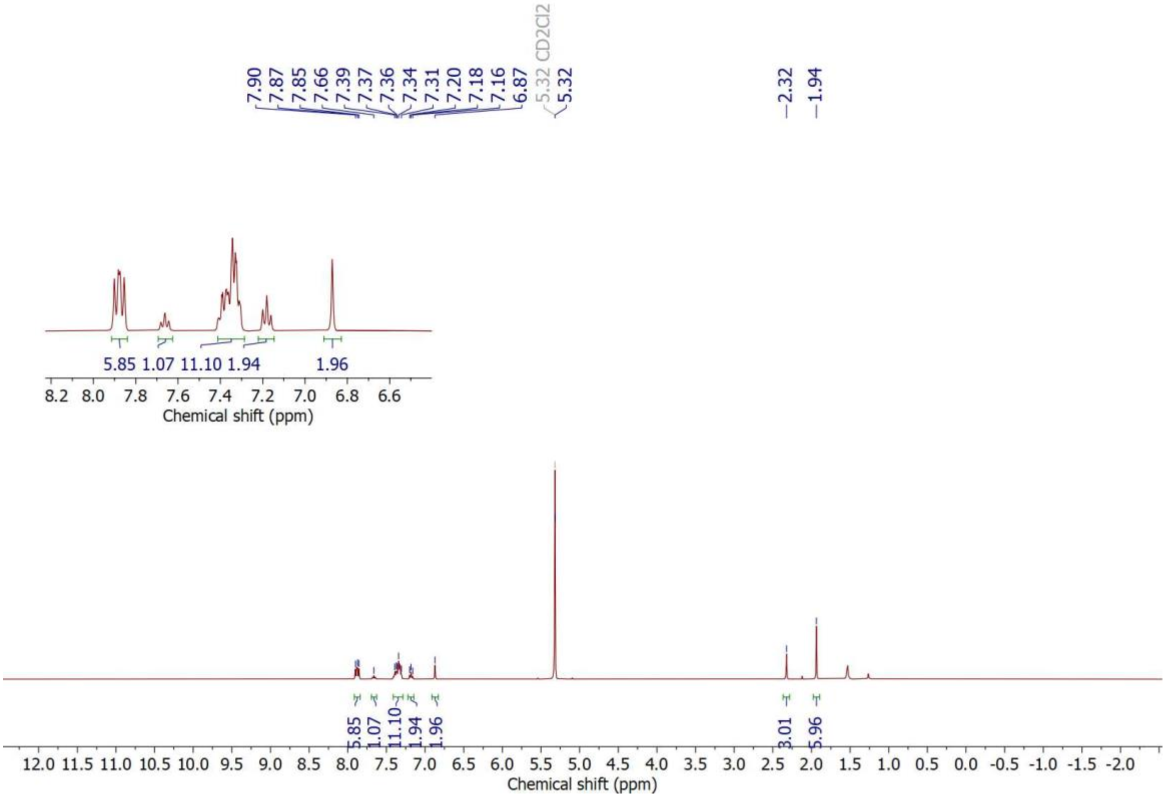

Figure S9: ^{1}H NMR spectrum of *fac*-[Tc(NPh)(PPh_3)(CNMes)Cl_3] (**2b**) in CD_2Cl_2.

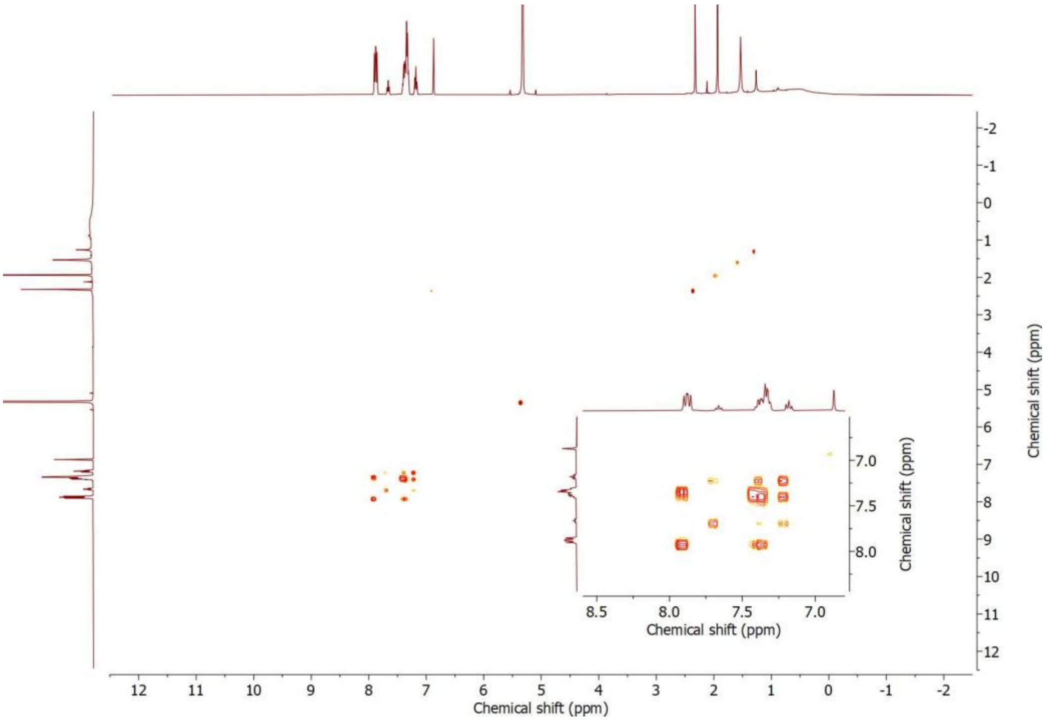

Figure S10: ^{1}H,^{1}H COSY NMR spectrum of *fac*-[Tc(NPh)(PPh_3)(CNMes)Cl_3] (**2b**) in CD_2Cl_2.

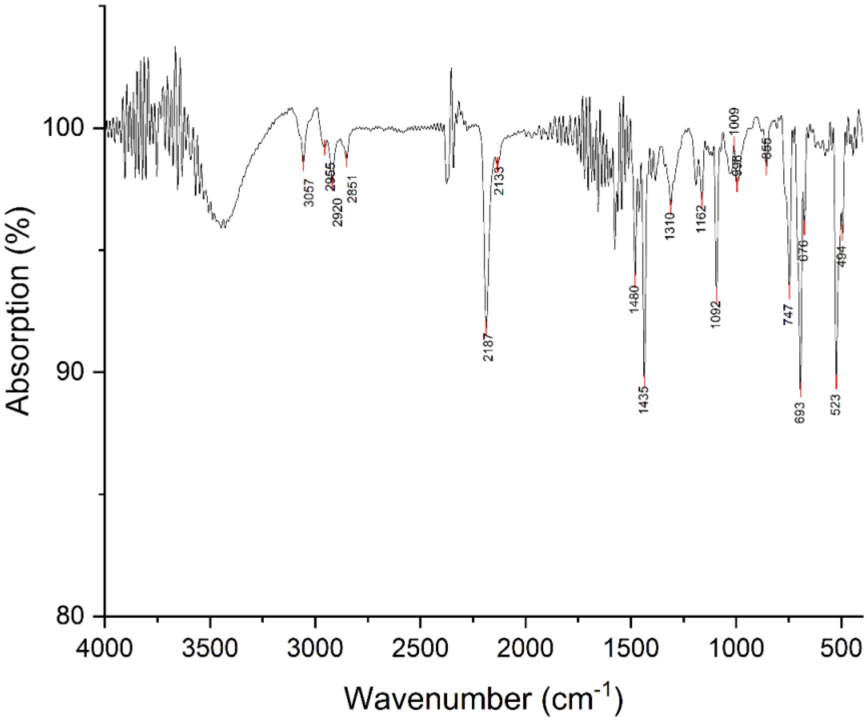

Figure S11: IR (KBr) spectrum of *fac*-[Tc(NPh)(PPh$_3$)(CNMes)Cl$_3$] (**2b**).

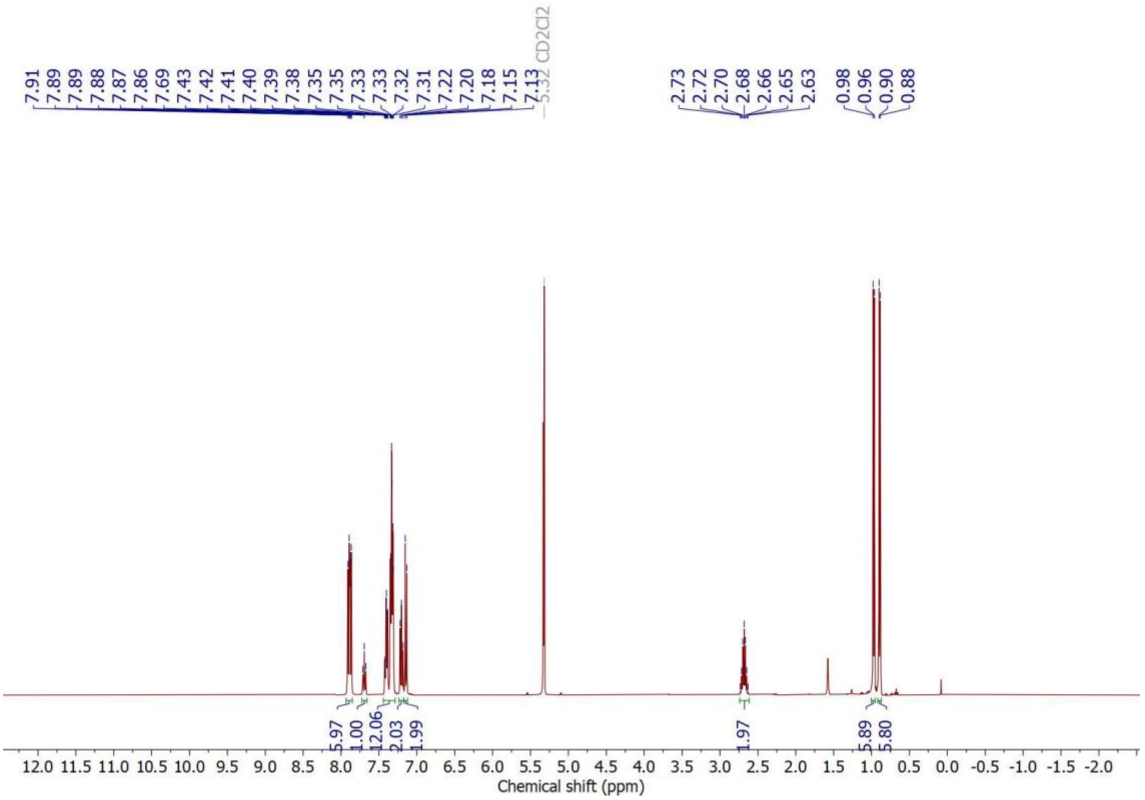

Figure S12: ^{1}H NMR spectrum of *fac*-[Tc(NPh)Cl$_3$(PPh$_3$)(CNPh$^{i\text{-}prop2}$)] (**2c**) in CD_2Cl_2.

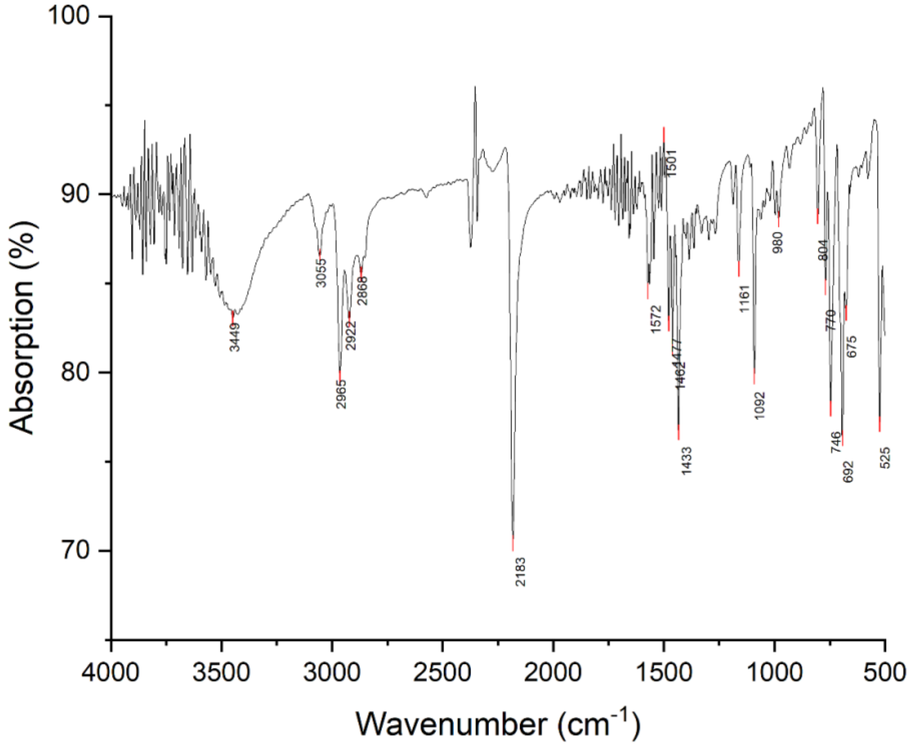

Figure S13: IR (KBr) spectrum of *fac*-[Tc(NPh)Cl_3(PPh_3)($CNPh^{i\text{-}prop2}$)] (**2c**).

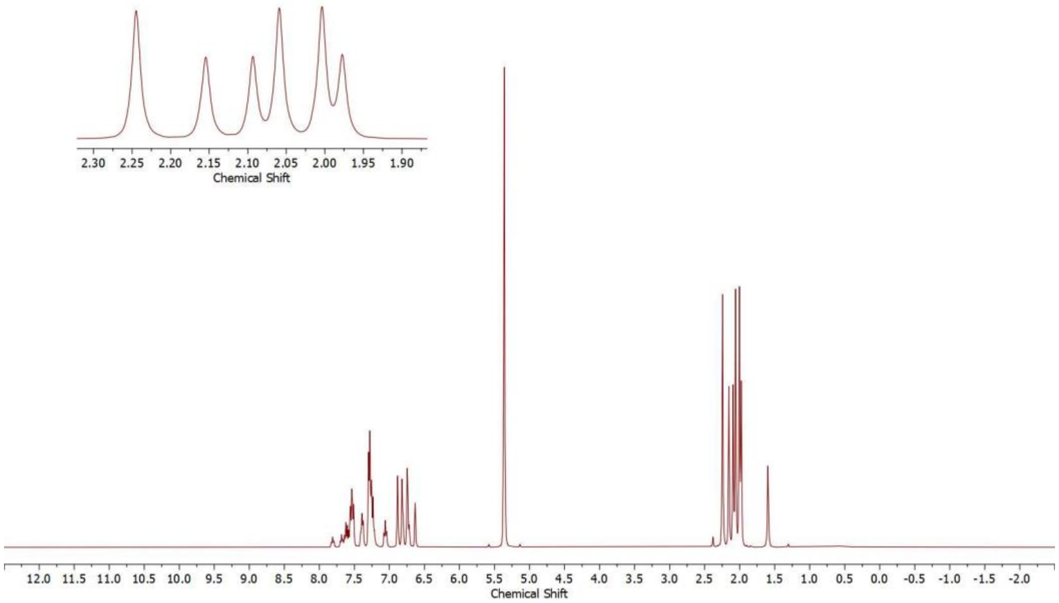

Figure S14: ^{1}H NMR spectrum of isomers of *fac*-[Tc(NPh)Cl_3(PPh_3)($CNAr^{Mes2}$)] (**3a**) in CD_2Cl_2.

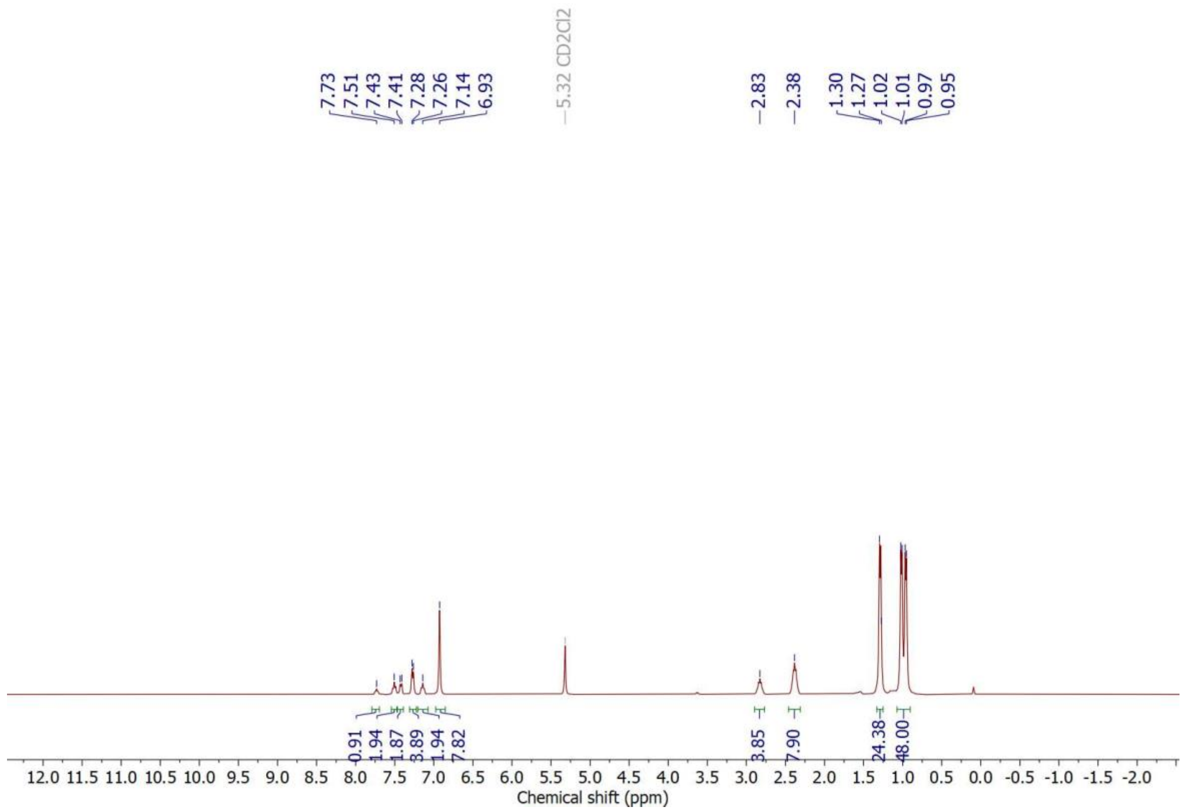

Figure S15: ^{1}H NMR spectrum of *trans,mer*-[Tc(NPh)Cl_3(CNAr^{Tripp2})$_2$] (**4b**) in CD_2Cl_2.

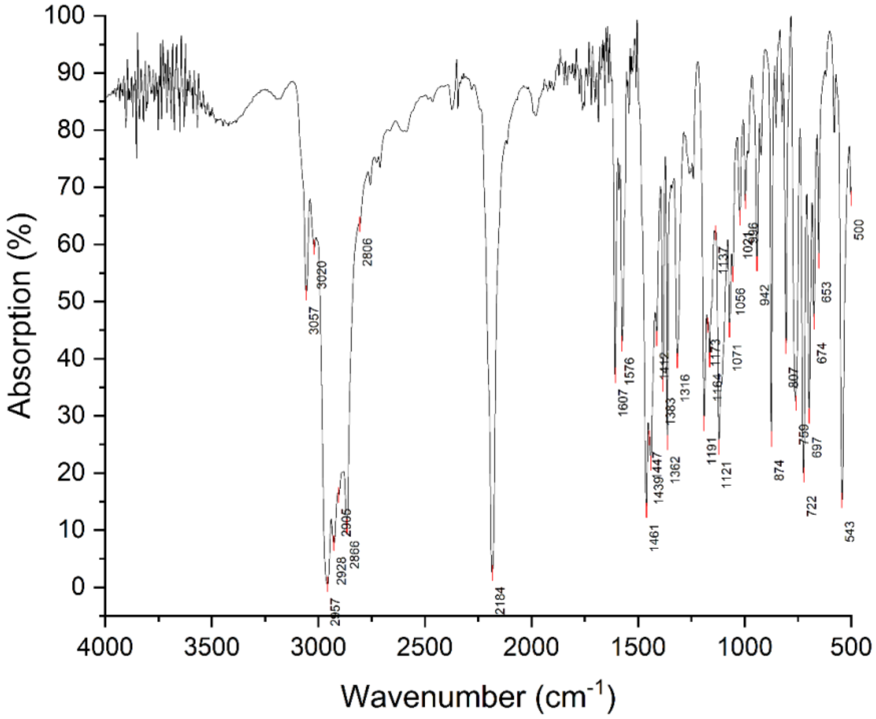

Figure S16: IR (KBr) spectrum of *trans,mer*-[Tc(NPh)(CNAr^{Tripp2})Cl_3] (**4b**).

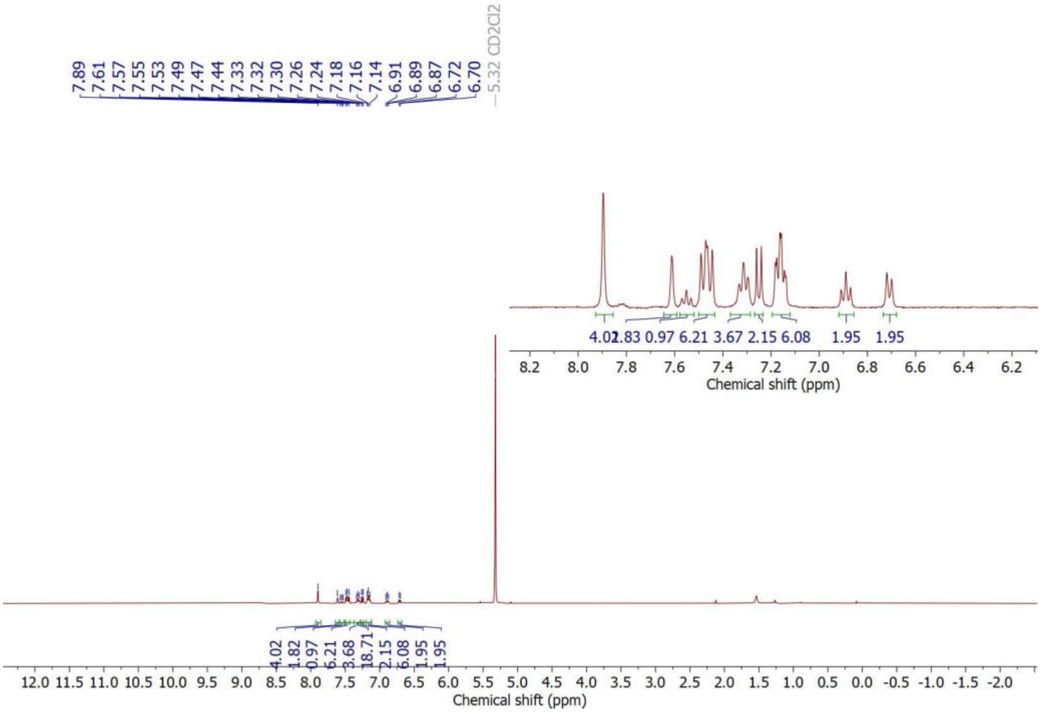

Figure S17: ^{1}H NMR spectrum of *fac*-[Tc(NPh)Cl_3(PPh_3)(CN*p*-FAr^{DarF2})] (**5**) in CD_2Cl_2.

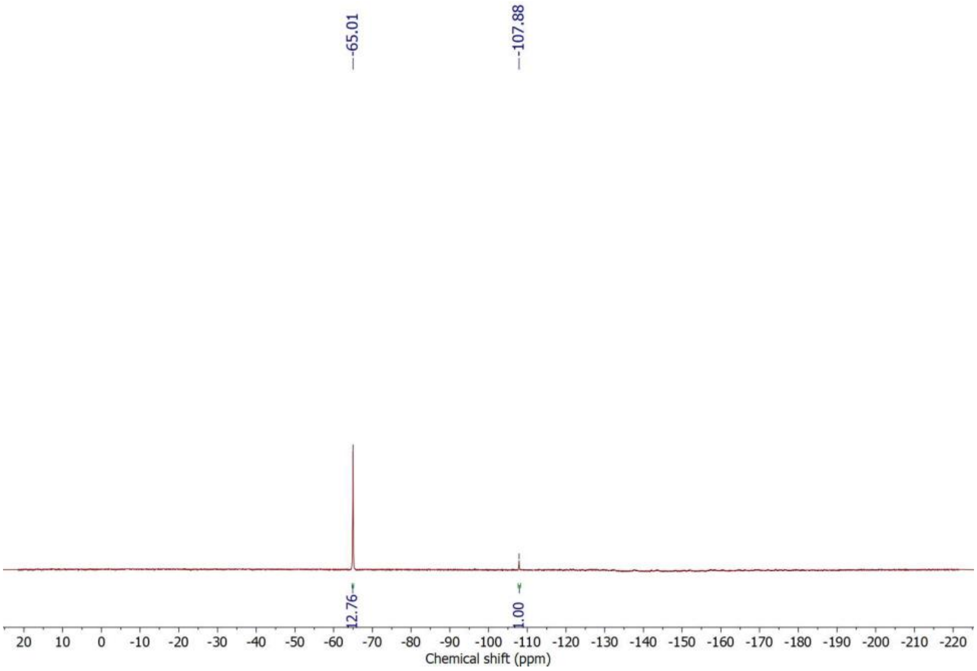

Figure S18: ^{19}F NMR spectrum of *fac*- [Tc(NPh)Cl_3(PPh_3)(CN*p*-FAr^{DarF2})] (**5**) in CD_2Cl_2.

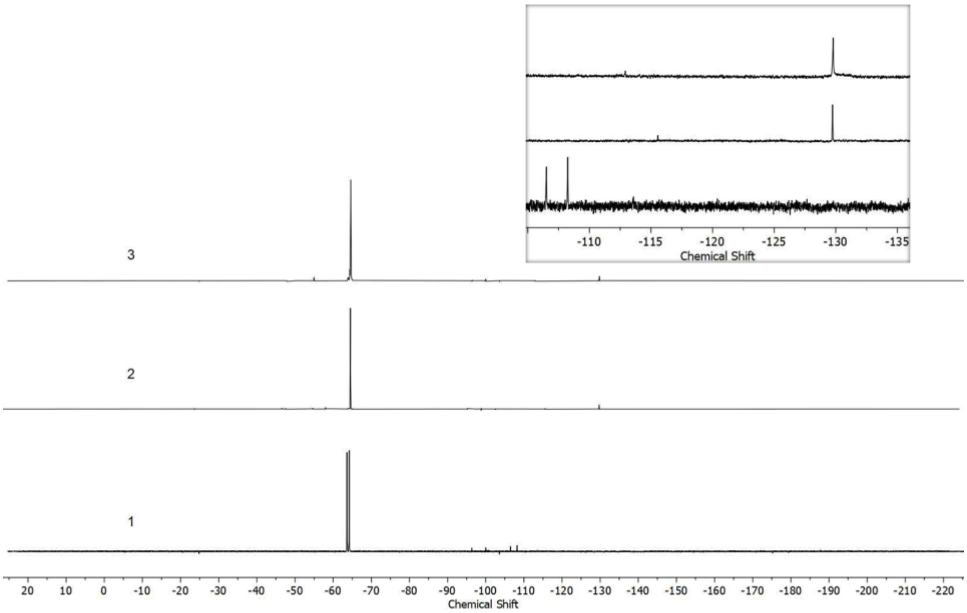

Figure S19: ^{19}F NMR spectra recorded during the reaction of *fac*-[Tc(NPh)Cl$_3$(PPh$_3$)(CN*p*-FAr^{DarF2})] (**5**) with CN*p*-FAr^{DarF2} in THF. Spectrum 1 was recorded after the addition of one equivalent of CN*p*-FAr^{DarF2} to **5**. Spectrum 2 was recorded after heating the previously obtained solution for 10 min in boiling THF. Spectrum 3 was recorded after heating a solution of complex **5** for 10 min in boiling THF.

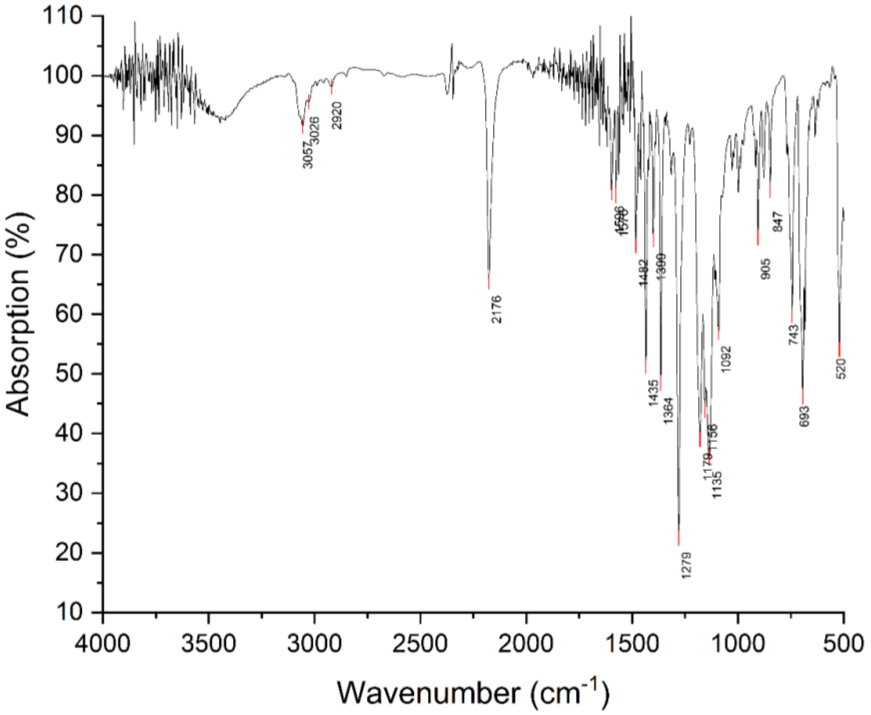

Figure S20: IR (KBr) spectrum of *fac*-[Tc(NPh)Cl$_3$(PPh$_3$)(CN*p*-FAr^{DarF2})] (**5**).

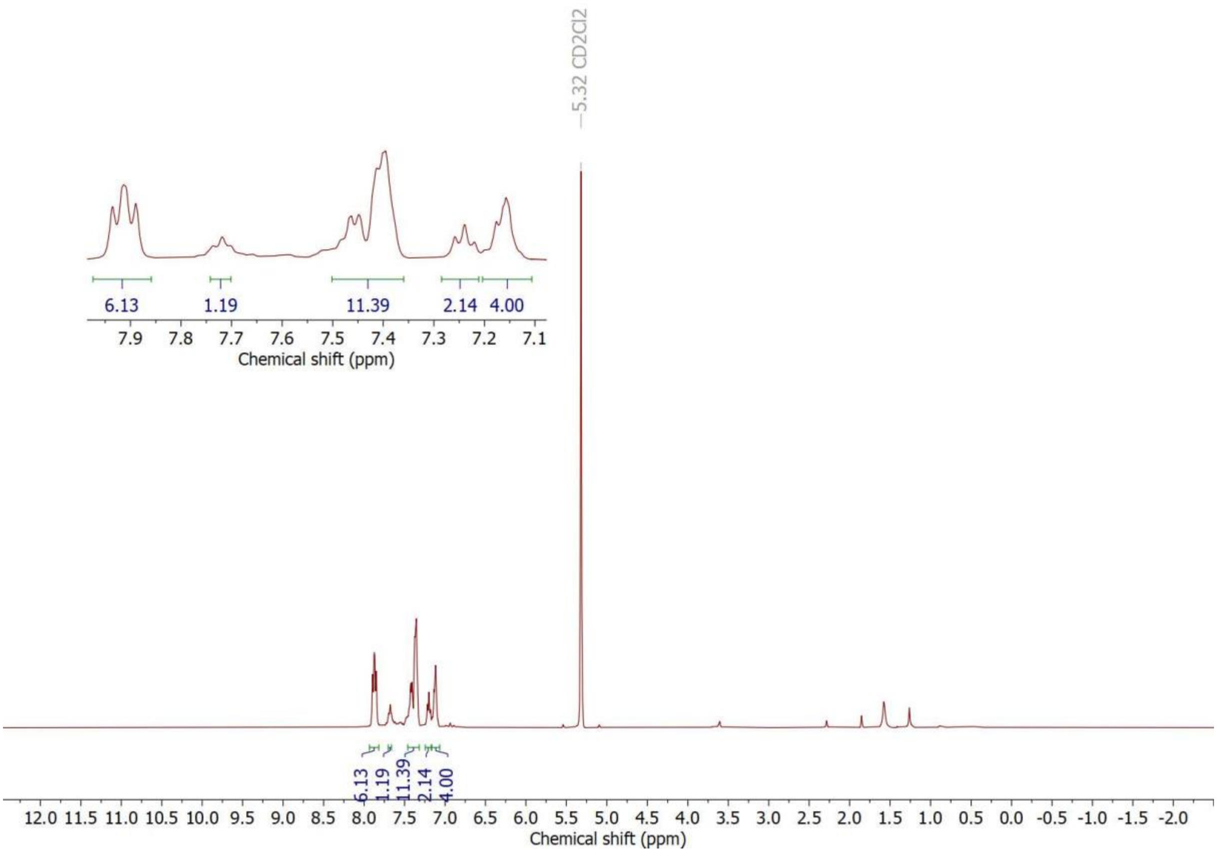

Figure S21: ^{1}H NMR spectrum of *fac*-[Tc(NPh)Cl_3(PPh_3)($CNPh^{pF}$)] (**6**) in CD_2Cl_2.

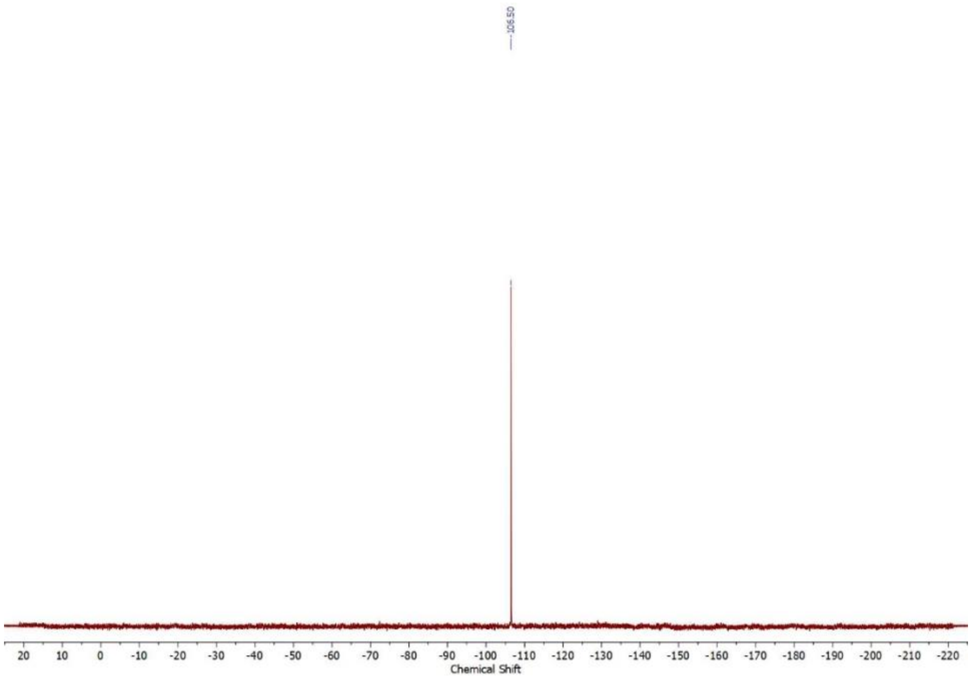

Figure S22: ^{19}F NMR spectrum of *fac*-[Tc(NPh)Cl_3(PPh_3)($CNPh^{pF}$)] (**6**) in CD_2Cl_2.

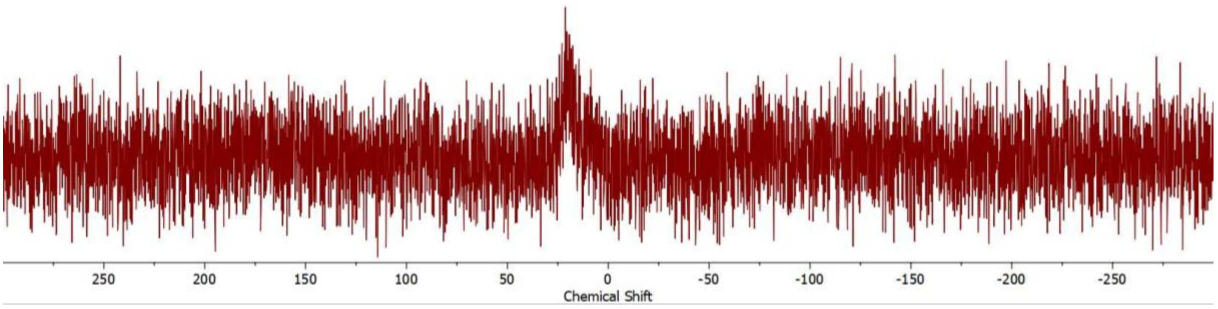

Figure S23: $^{31}P\{^{1}H\}$ NMR spectrum of *fac*-[Tc(NPh)Cl_3(PPh_3)($CNPh^{pF}$)] (**6**) in CD_2Cl_2.

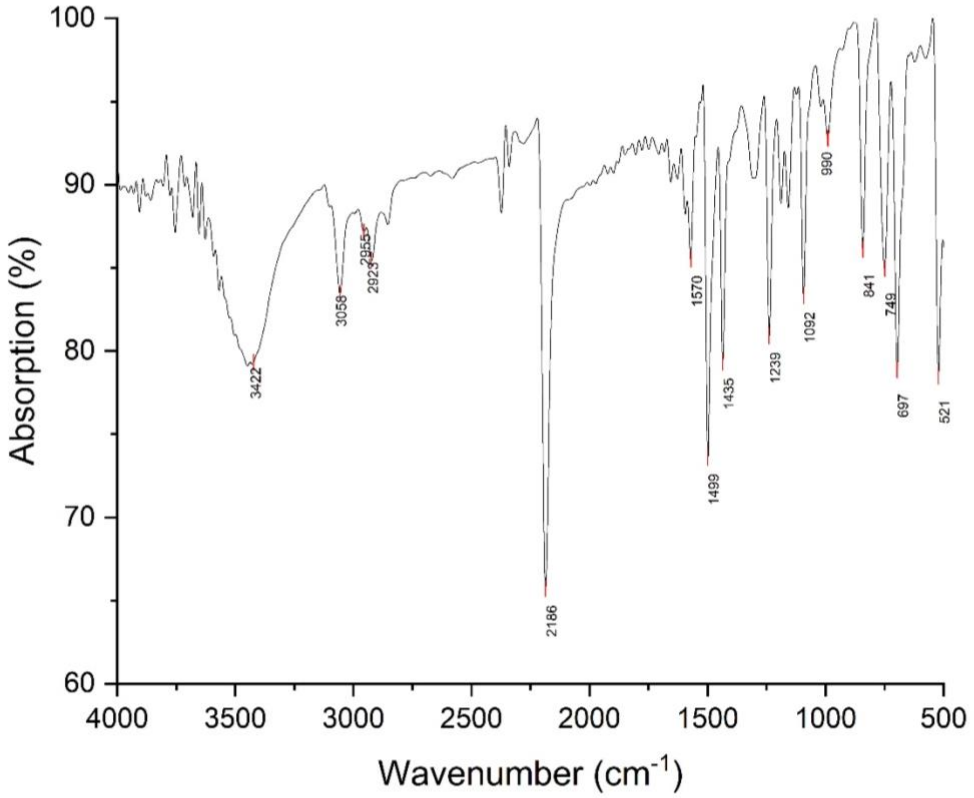

Figure S24: IR (KBr) spectrum of *fac*-[Tc(NPh)Cl_3(PPh_3)($CNPh^{pF}$)] (**6**).

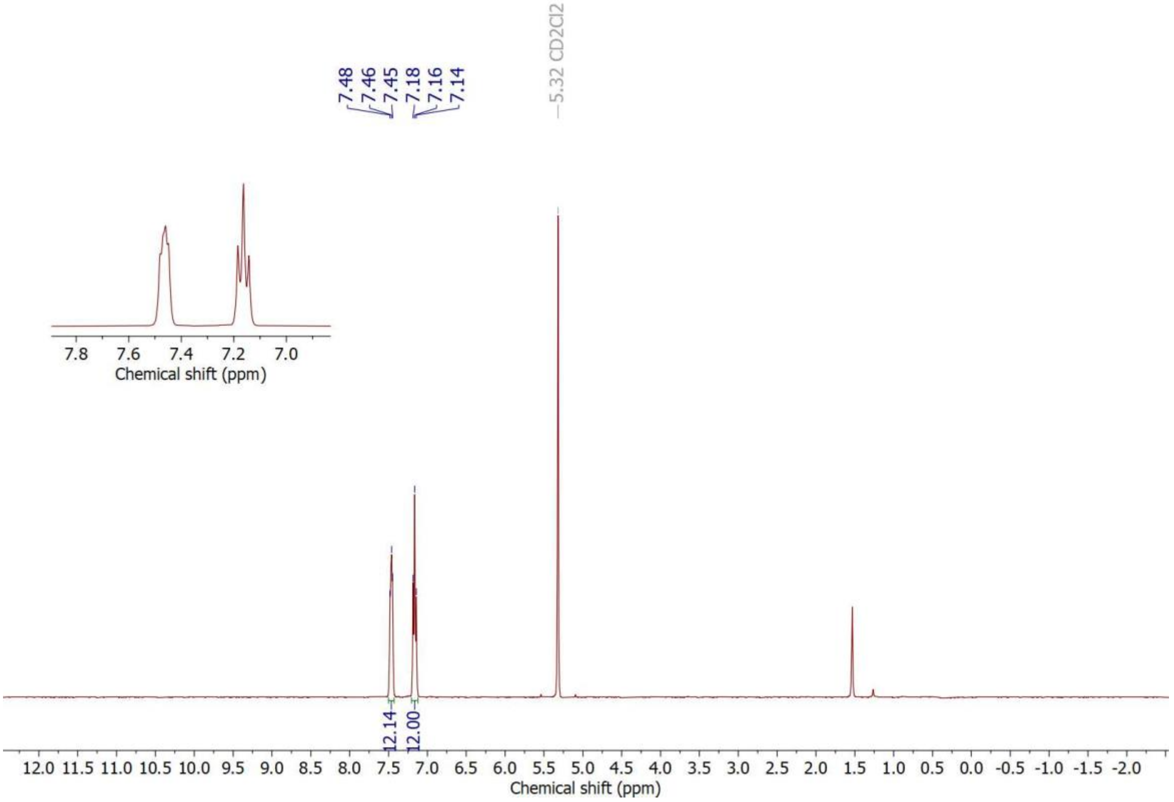

Figure S25: ^{1}H NMR spectrum of *fac*-[Tc(CNPh^{pF})$_6$][PF$_6$] (**7**) in CD_2Cl_2.

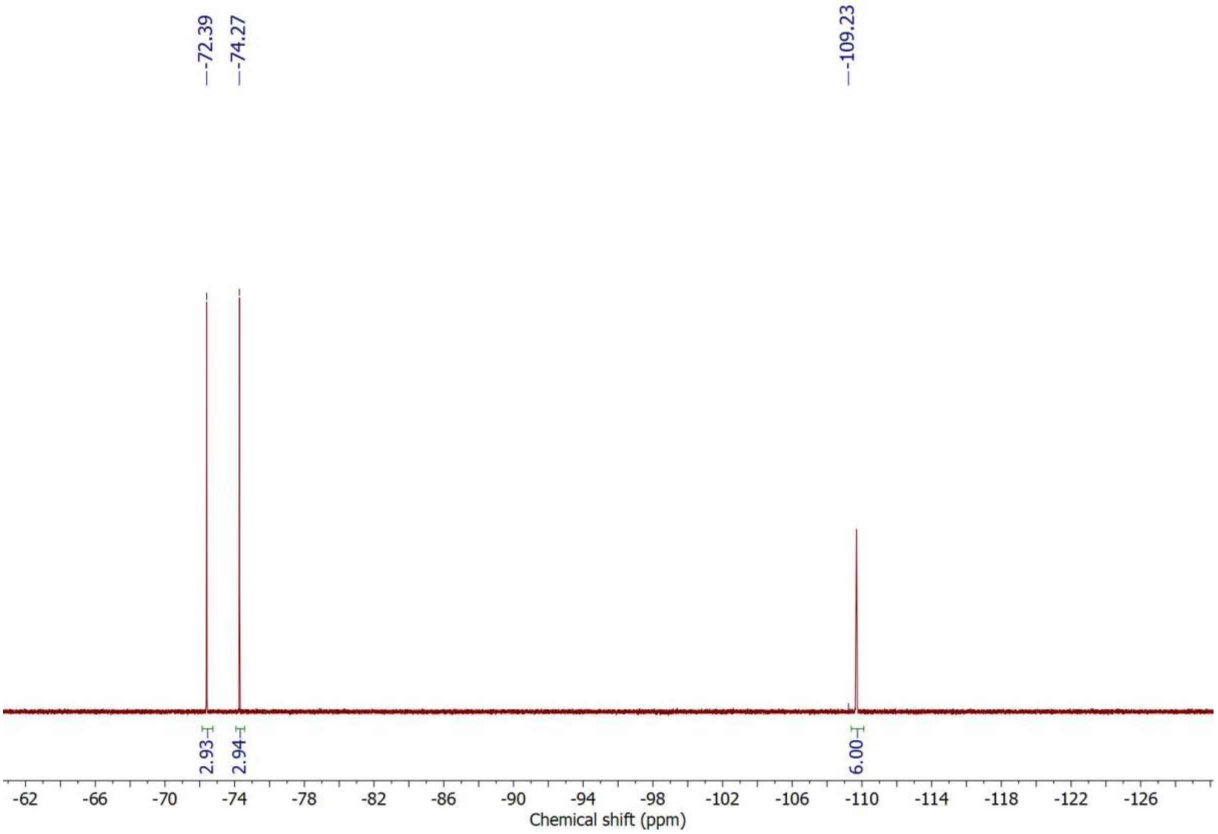

Figure S26: ^{19}F NMR spectrum of [Tc(CNPh^{pF})$_6$][PF$_6$] (**7**) in CD_2Cl_2.

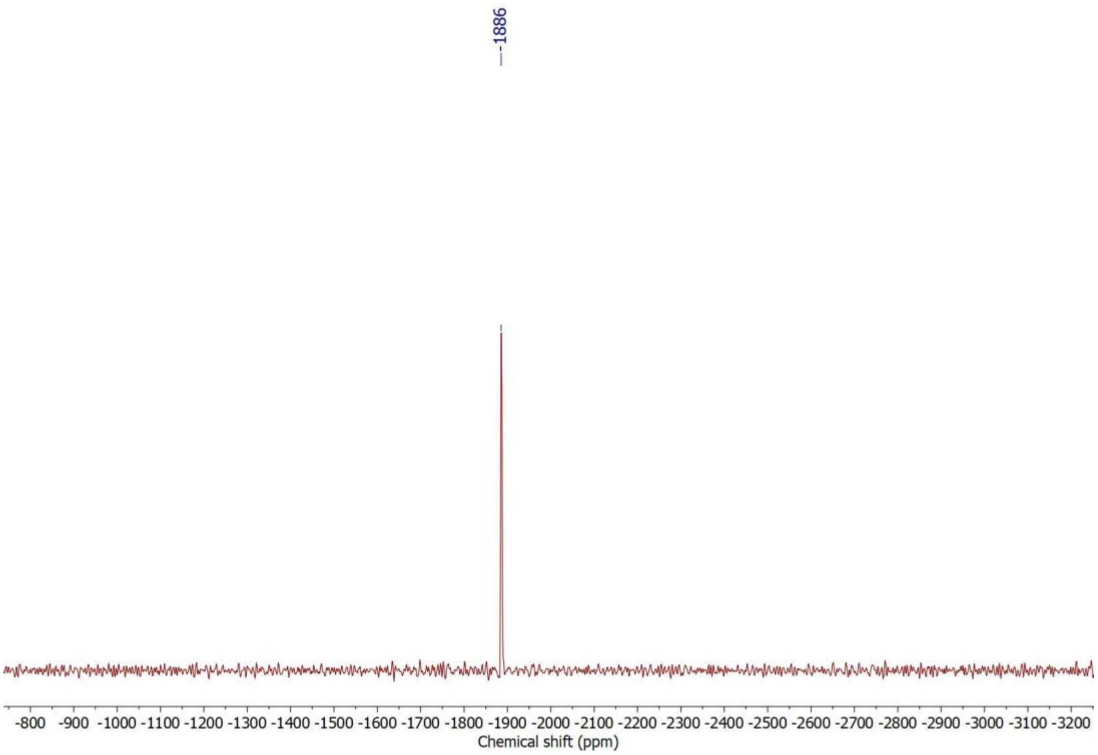

Figure S27: ^{99}Tc NMR spectrum of $[Tc(CNPh^{pF})_6][PF_6]$ (**7**) in CD_2Cl_2.

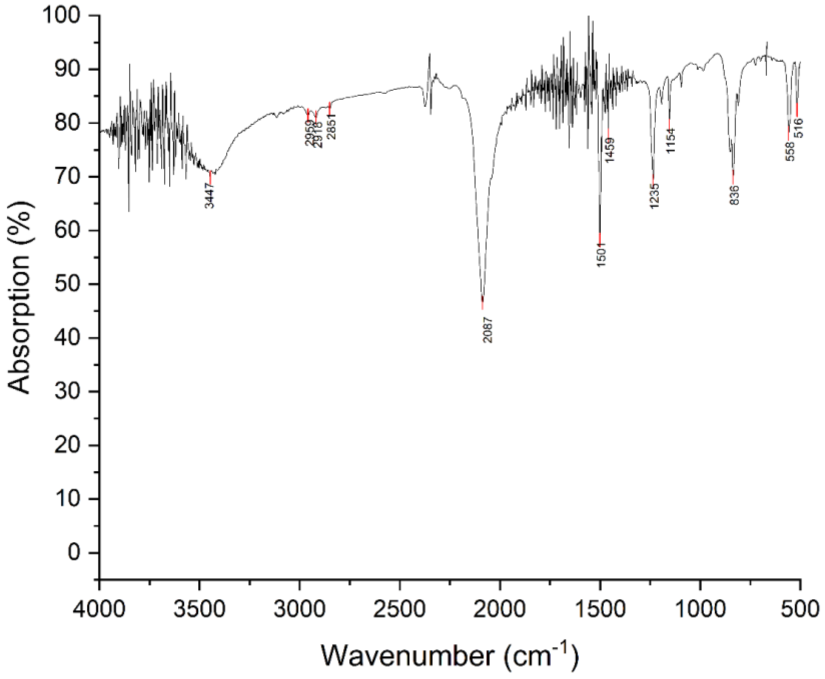

Figure S28: IR (KBr) spectrum of $[Tc(CNPh^{pF})_6][PF_6]$ (**7**).

DFT Calculations

Table S8. Calculated electrostatic potential surface properties of the isocyanide carbon atom at the Van der Waals (VdW) boundary for structures optimized at the B3LYP/6-311++G** level. Surface properties were evaluated at $\rho = 0.001$ level using an electrostatic potential map basis with a grid-point spacing of 0.25. The last column contains the Surface-Averaged Donor Atom Potential SADAP = (EP_{min} + EP_{max} + AP)/(ES_{pos} + ES_{neg}) as a combined descriptor of steric and electrostatic properties of the potential ligands, which allows an estimation of their reactivity.

CN-R	Exposed VdW surface, ES (Å^2)		Extrema for potential energies at VdW surface, EP (kcal/mol)		Average potential energies at VdW surface, AP (kcal/mol)	Surface-averaged donor atom potential SADAP, (kcal/mol Å^2)
R =	ES_{pos}	ES_{neg}	EP_{min}	EP_{max}	$\varnothing_{overall}$	SADAP
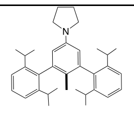 CNAr$^{(DippNC4H8)}$	0.00	21.81	-42.74	-15.96	-31.70	-4.15
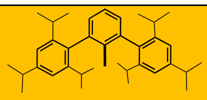 TRIP	0.00	22.23	-38.01	-9.31	-26.65	-3.33
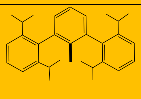 CNAr^{Dipp2}	0.00	22.13	-37.64	-8.87	-26.04	-3.28
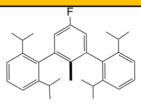 CNp-FAr^{Dipp2}	0.00	22.14	-36.22	-7.19	-24.42	-3.06
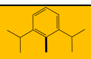 CNPh$^{i\text{-}prop2}$	0.00	25.99	-35.47	-5.16	-21.20	-2.38
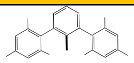 CNAr^{Mes2}	0.00	30.10	-39.20	-7.01	-25.11	-2.37
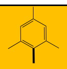 CNMes	0.00	28.89	-36.79	-6.83	-21.68	-2.26
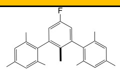 CNp-FAr^{Mes2}	0.00	30.13	-37.67	-5.04	-23.31	-2.19
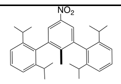 CNp-NO_2Ar^{Dipp2}	0.01	22.81	-31.40	0.48	-18.83	-2.18

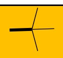 CN^{t}Bu	0.00	31.43	-39.63	-5.86	-22.00	-2.15
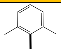 CNPh$^{o,o\text{-}Me2}$	0.00	28.80	-35.60	-5.35	-20.34	-2.13
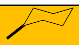 CNCy	0.00	31.61	-39.23	-5.09	-21.81	-2.09
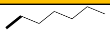 CN^{n}Hex	0.00	31.61	-38.62	-5.69	-21.48	-2.08
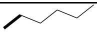 CN^{n}Pent	0.00	31.58	-38.60	-5.67	-21.45	-2.08
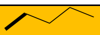 CN^{n}Bu	0.00	31.58	-38.51	-5.52	-21.36	-2.07
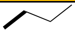 CN^{n}Pr	0.00	31.57	-38.37	-5.51	-21.23	-2.06
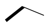 CNEt	0.00	31.62	-38.06	-5.17	-20.98	-2.03
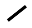 CNMe	0.00	31.52	-37.41	-6.01	-20.50	-2.03
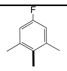 CNPh$^{o,o\text{-}Me2,p\text{-}F}$	0.00	28.79	-33.68	-2.45	-18.02	-1.88
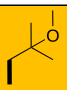 MIBI	0.02	29.07	-35.96	2.30	-18.65	-1.80
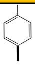 CNPh$^{p\text{-}Me}$	0.01	31.24	-35.69	0.94	-18.57	-1.71
H CNH	0.00	31.12	-31.48	-4.62	-16.42	-1.69
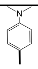 CNPh$^{p\text{-}NMe2}$	0.16	31.28	-36.06	2.15	-18.61	-1.67
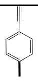 CNPh$^{p\text{-}CCH}$	0.15	30.08	-34.98	1.79	-17.21	-1.67
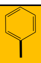 CNPh	0.05	31.28	-35.20	1.72	-18.01	-1.64

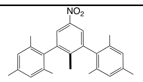 CNp-NO_2Ar^{Mes2}	0.17	29.91	-32.56	2.46	-17.50	-1.58
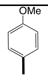 CNPh$^{p\text{-}OMe}$	0.43	30.95	-34.17	4.50	-16.60	-1.47
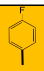 CNPh$^{p\text{-}F}$	1.67	29.53	-32.02	7.05	-14.10	-1.25
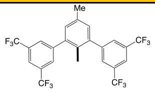 CNp-MeAr^{DArF2}	1.87	26.11	-30.32	9.17	-13.34	-1.23
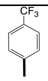 CNPh$^{p\text{-}CF3}$	1.86	29.19	-31.18	6.80	-13.37	-1.22
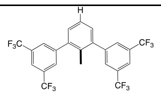 CNAr^{DArF2}	3.43	24.42	-29.04	10.22	-11.95	-1.10
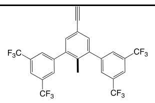 CN(p-CCH)Ar^{DArF2}	4.52	23.37	-28.26	11.17	-11.03	-1.01
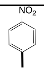 CNPh$^{p\text{-}NO2}$	6.15	24.86	-28.59	9.44	-10.27	-0.95
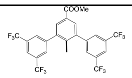 CN(p-COOMe)Ar^{DArF2}	5.45	22.34	-27.45	12.53	-10.13	-0.90
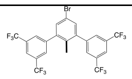 CNp-BrAr^{DArF2}	5.61	22.28	-27.38	12.50	-10.03	-0.89
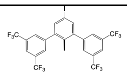 CNp-IAr^{DArF2}	5.61	22.34	-27.42	12.53	-10.04	-0.89
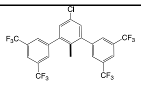 CNp-ClAr^{DArF2}	5.74	22.15	-27.30	12.92	-9.96	-0.87
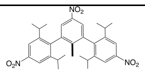 CN(p-NO_2)Ar$^{(p\text{-}NO2Dipp)2}$	4.92	18.14	-23.28	12.74	-8.26	-0.81

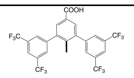 CN(p-COOH)Ar^{DArF2}	6.74	21.13	-26.39	14.75	-8.89	-0.74
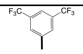 $CNPh^{m,m-(CF3)2}$	8.23	22.96	-27.55	14.48	-8.68	-0.70
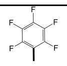 $CNPh^{F5}$	9.36	21.48	-25.27	14.24	-7.07	-0.59
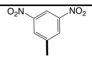 $CNPh^{m.m-(NO2)2}$	12.09	19.09	-23.33	14.43	-4.67	-0.44
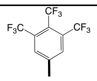 $CNPh^{p,m,m-(CF3)3}$	11.49	19.44	-24.62	17.89	-5.28	-0.39
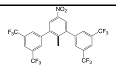 CNp-NO_2Ar^{DArF2}	10.54	17.20	-22.73	18.30	-4.73	-0.33
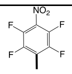 $CNPh^{p-NO2,F4}$	12.46	18.27	-22.28	18.10	-3.77	-0.26
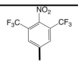 $CNPh^{p-NO2,m,m-(CF3)2}$	14.33	17.03	-21.39	17.29	-2.32	-0.20
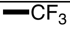 CNCF3	15.06	15.57	-19.94	16.88	-0.94	-0.13
CO	19.29	10.31	-12.49	12.42	3.35	0.11
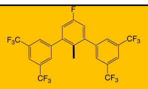 CNp-FAr^{DArF2}	20.50	6.74	-11.49	69.07	16.88	2.73

Table S9. LP_C/π^*_{CN} properties of some representative, free isocyanides, number of donated σ_{CTc} electrons ($\#e^-$; $LP_C \rightarrow LP^*_{Tc}$) and second order perturbation parameters (interaction energy E; energy difference between the two orbitals E_i-E_j; overlap parameter A) for $LP_{Tc} \rightarrow \pi^*_{CN}$ in the model complexes $[TcO_3(CO/CNR)]^+$ and $[Tc(CO)_5(CO/CNR)]^+$ (R = Ph^{F5}, $Ph^{p\text{-}F}$, Ph, ^{t}Bu, Ar^{DArF2}).

				$[TcO_3(L)]^+$	$[Tc(CO)_5(L)]^+$			
L	π^*_{CN}	LP_C	$\Delta(\pi^*_{CN}\text{-}LP_C)$	$\#e^-/\sigma_{CTc}$	$\#e\text{-}/\sigma_{CTc}$	$E(LP_{Tc} \rightarrow \pi^*_{CN})$ [kJ/mol]	E_i-E_j [a.u.]	A
$CNAr^{Dipp2}$	-1.53	-8.48	6.95					
CN^tBu	2.74	-11.15	13.89	1.9029	1.8910	8.37/8.37	0.32/0.32	0.049/0.049
CNPh	2.20	-11.59	13.80	1.9033	1.8923	9.00/9.06	0.31/0.31	0.050/0.049
$CNPh^{p\text{-}F}$	2.04	-11.72	13.76	1.9035	1.8927	9.13/9.17	0.31/0.31	0.050/0.051
$CNPh^{F5}$	1.48	-12.36	13.84	1.9076	1.8947	10.64/10.62	0.30/0.30	0.054/0.052
$CNAr^{DArF2}$	103.58	-12.18	115.76	1.9022	1.8919	9.49/9.55	0.31/0.31	0.052/0.050
CO	-1.16	-10.55	9.39	1.9364	1.9111	13.81/13.81	0.28/0.28	0.059/0.059
